# Retrotransposition and mutation events yield Rap1 GTPases with differential signalling capacity

**DOI:** 10.1186/1471-2148-10-55

**Published:** 2010-02-19

**Authors:** Tomasz Zemojtel, Marlena Duchniewicz, Zhongchun Zhang, Taisa Paluch, Hannes Luz, Tobias Penzkofer, Jürgen S Scheele, Fried JT Zwartkruis

**Affiliations:** 1 Department of Computational Molecular Biology, Max Planck Institute for Molecular Genetics, Ihnestrasse 73, D-14195 Berlin, Germany; 2 Department of Bioinformatics, University of Würzburg, Am Hubland, D-97074 Würzburg, Germany; 3 Helmholtz-Institute for Biomedical Engineering, RWTH Aachen University, Aachen, Germany; 4 Department of Medicine I, University of Freiburg Medical Center, D-79106 Freiburg, Germany; 5 Department of Physiological Chemistry, Center for Biomedical Genetics and Cancer Genomics Centre, University Medical Centre Utrecht, Universiteitsweg 100, 3584 CG Utrecht, The Netherlands

## Abstract

**Background:**

Retrotransposition of mRNA transcripts gives occasionally rise to functional retrogenes. Through acquiring tempero-spatial expression patterns distinct from their parental genes and/or functional mutations in their coding sequences, such retrogenes may in principle reshape signalling networks.

**Results:**

Here we present evidence for such a scenario, involving retrogenes of Rap1 belonging to the Ras family of small GTPases. We identified two murine and one human-specific retrogene of Rap1A and Rap1B, which encode proteins that differ by only a few amino acids from their parental Rap1 proteins. Markedly, human hRap1B-retro and mouse mRap1A-retro1 acquired mutations in the 12^th ^and 59^th ^amino acids, respectively, corresponding to residues mutated in constitutively active oncogenic Ras proteins. Statistical and structural analyses support a functional evolution scenario, where Rap1 isoforms of retrogenic origin are functionally distinct from their parental proteins. Indeed, all retrogene-encoded GTPases have an increased GTP/GDP binding ratio *in vivo*, indicating that their conformations resemble that of active GTP-bound Rap1. We furthermore demonstrate that these three Rap1 isoforms exhibit distinct affinities for the Ras-binding domain of RalGDS. Finally, when tested for their capacity to induce key cellular processes like integrin-mediated cell adhesion or cell spreading, marked differences are seen.

**Conclusions:**

Together, these data lend strong support for an evolution scenario, where retrotransposition and subsequent mutation events generated species-specific Rap1 isoforms with differential signaling potential. Expression of the constitutively active human Rap1B-retro in cells like those derived from Ramos Burkitt's lymphoma and bone marrow from a patient with myelodysplastic syndrome (MDS) warrants further investigation into its role in disease development.

## Background

Development of new functional genes is central to the evolution of species-specific traits [1]. Two major mechanisms allow for creation of new genes based on existing templates: duplication of chromosomal regions containing gene loci and retrotransposition of mRNA transcripts into the genome - a process which in mammals is facilitated by active LINE-1 retrotransposons (L1s) [2,3]. Novel functions for processed genes, termed retrogenes, may come from the acquirement of new spatio-temporal expression patterns, dictated by the genomic context of cDNA insertion [4-8]. Furthermore, functional evolution of their regulatory sequences and coding sequences, driven by natural selection, could result in new functions within signaling networks [5,9,10].

Evidence for the latter mode of gene evolution is scarce. First of all, expression of retrogenes has only recently begun to be explored. Although expression of processed genes seems to be more common than previously anticipated, surveys are frequently limited to a single species [9-11]. In addition, the physiological consequences of altered gene expression patterns [4,5,12] and/or the biochemical consequences of amino acid substitutions are rarely investigated [1,5].

In the present study, we provide evidence for the functional evolution of retrogenes derived from the Ras-like GTPase, Rap1. The activity of Rap1 is dynamically controlled by many different receptor proteins, including tyrosine receptor kinases and G-protein coupled receptors [13]. These receptors regulate the activity of guanine nucleotide exchange factors (GEFs), resulting in GTP-binding of Rap1, or GTPase activating proteins (GAPs) that strongly increase the hydrolyzing capacity of Rap1 and thereby inactivate Rap1 [14]. Rap1 is involved in many different morphogenetic processes e.g. by affecting the activity of cell-matrix or cell-cell adhesion receptors. In addition, Rap1 plays important physiological roles in established structures like for example the vertebrate blood vessel system. Here, an acute rise in cAMP results in immediate enhancement of the level of GTP-bound Rap1 that is crucial for an increase in vascular permeability [15].

Rap1A and Rap1B are paralogs and differ only by 9 amino acids, that are mostly located at the very C-terminal part [16]. Rap1 proteins are highly conserved in evolution and in fact identical Rap1A as well as Rap1B proteins are found in human, chimp, macaque, cow and mouse. While both proteins are ubiquitously expressed in tissues, a differential distribution of isoforms has been observed, e.g. Rap1B protein is predominant in platelets [17]. Possibly, the high level of sequence conservation and differential gene expression reflects specific functions for each of these GTPases. This would be consistent with the outcome of studies in mice with targeted disruption of Rap1 genes. Loss of Rap1B impairs platelet aggregation due to a diminished activation of α_IIb_β_3 _integrins. In addition, defects in endothelial cells may cause bleedings, resulting in a high percentage of embryonic lethality [18]. Rap1A knockout mice are fully viable and fertile, but haematopoietic cells from the spleen and thymus adhere less efficiently to fibronectin or collagen as compared to wild type cells [19]. In agreement with earlier *in vitro *observations [20], a recent work reported a reduced superoxide production in Rap1A -/- neutrophils [21]. Double Rap1A/Rap1B knockout mice die during early embryogenesis suggesting functional overlap between the two Rap1 isoforms [22].

Our current study suggests that additional complexity resides within the Rap1 signaling network. We characterize here two murine-specific Rap1A retrogenes and one human-specific Rap1B retrogene that acquired limited substitutions in amino acids, including the residues G12 and A59 that were found to be mutated in oncogenic Ras isoforms. By using computational and experimental approaches we collected evidence that the species-specific events of retrotransposition and mutation created new Rap1 isoforms whose signaling capacity differs from that of their parental isoforms. We discuss potential roles for Rap1 retrogenes in cell physiology.

## Results

### Identification of Rap1A and Rap1B retrogenes with limited mutations

**Figure 1:**
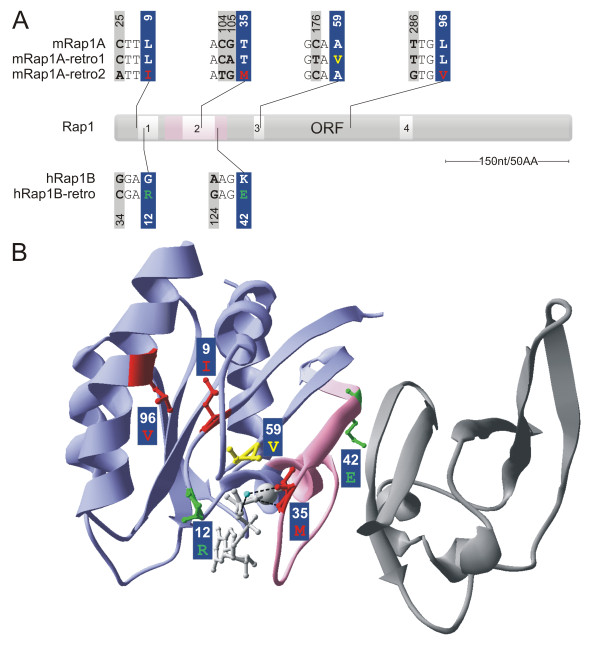
**Comparison of the mouse and human Rap1 retrogenes**. **A**. Overview of nucleotide changes in the open reading frame (ORF) regions of the three retrogenes. Changes in amino acid sequence are highlighted in three different colors on the blue background, corresponding to each of the retrogenes (Rap1A-retro1 in yellow; Rap1B-retro2 in red; hRap1B-retro in green). mRap1A, mouse Rap1A sequence (NM_145541); hRap1B, human Rap1B sequence (NM_015646). 1-4 in the ORF indicate the conserved motifs, G1-4, that bind the nucleotides and/or interact with effector proteins (G1: ^10^**G**xxxx**GKS**^17^, Walker A; G2: ^28^**FV**xx**Y**x**PT**xx**D**x**Y**^40^, G3: ^57^**D**xx**G**^60^; G4: ^116^**NK**x**D**^119^). The effector domain region is marked in pink (amino acids 20-45: 32-40 the general recognition motif; and 20-31, 41-45 specificity regions, [33]). **B**. Locations of retrogene-specific amino acid substitutions mapped onto the structure of Rap1A in complex with the Ras-binding domain (RBD) of c-Raf1 (PDB: 1C1Y). Hydrogen bounds between the T35 residue and, GTP and the water molecule are highlighted with black dashed lines. The water molecule is marked in light-blue, GTP in light-gray, Mg2+ is represented as a large light-gray ball.

We have recently generated a Rap1A-deficient mouse strain [19]. Unexpectedly, PCR on cDNA derived from tissues of Rap1A -/- mice with a 5' primer recognizing the targeted second exon sequence and a 3' primer located in the last exon yielded products, thus suggesting the existence of another mRNA homologous to Rap1A. Indeed, we cloned two cDNAs that showed high homology to mouse Rap1A (mRap1A), yet encoded proteins with either a single or triple amino acid substitution as indicated in Figure 1A and 1B. We named these genes mRap1A-retro1 (containing the mutation A59V) and mRap1A-retro2 (I9L, T35M, L96V). Based on mutational and structural analysis of Ras-like GTPases, it seemed likely that mRap1A-retro1 and mRap1A-retro2 would encode for activated (GTP-bound) versions of Rap1A.

**Figure 2:**
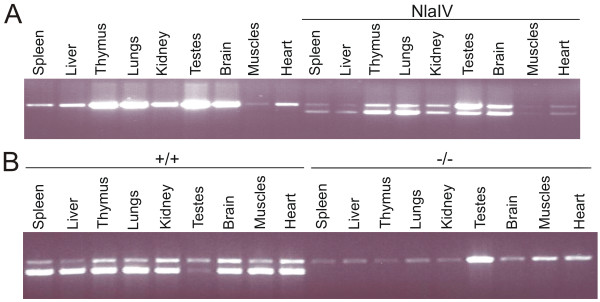
**RT PCR reaction of mRap1A and mRap1A-retro1, mRap1A-retro2 mRNA**. A. RT-PCR reaction with RNA isolated from WT organs. One half of RT-PCR reaction mixture was directly applied on gel (lines 1-9) while the second half was first digested with NlaIV (lines 10-18). B. RT-PCR product was digested with NlaIV restriction enzyme and loaded on the gel. Lanes 1 to 9 represent samples from wt organs (+/+) and lanes 10 to 18 represent organs of Rap1A deficient mice (-/-).

Remarkably, database searches did not identify any mouse ESTs corresponding to either of the mRap1A-retro transcripts. To establish that the cloned mRap1A-retro1 and mRap1A-retro2 were not derived from amplification of genomic DNA, two control experiments were done. First, reverse transcriptase was left out of the RT-PCR reaction, which abolished amplification of the cDNAs. Secondly, the cDNAs were amplified using the Gene Racer kit, which relies on removal of the CAP from mRNA and subsequent ligation of a PCR primer to the now available phosphate group at the 5' end of the mRNA (see Methods). The RNA expression of both retrogenes was analysed in WT and Rap1A -/- organs by RT-PCR. Using PCR primers (Methods), which allow for simultaneous amplification of mRap1A-retro1, -retro2 and mRap1A mRNAs, a single PCR product of 1186 bp comprising the complete coding sequence and a large part of 3'UTR, was amplified (Figure 2A). We identified a NlaIV restriction site unique for the mRap1A cDNA and used it to distinguish between transcripts of mRap1A-retro1, -retro2 and that of mRap1A. Digestion of the 1186 bp PCR fragment of mRap1A yielded two fragments of 213 and 973 bp. By means of this approach we could detect expression of mRap1A-retro1 and mRap1A-retro2 in multiple tissues like thymus, lungs, testis and brain (Figure 2). Importantly, when RT-PCR was done on Rap1A -/- tissue with one primer placed in the deleted second exon (see Methods) cDNAs of both retrogenes were amplified (Figure 2B and data not shown). The same was observed, when primers specific for Rap1A retrogenes were used [Additional file 1: Supplemental Figure S1] (see Methods).

Importantly, we previously pointed out that also in human there is evidence for expression of a Rap1B retrogene (G12R, K42E), named hRap1B-retro [23]. More specifically, database searches revealed expression of this retrogene in Ramos Burkitt's lymphoma, neuroblastoma cell lines and placental tissue, while a study by Gyan et al. detected expression in a bone marrow sample from a patient with myelodysplastic syndrome (MDS) [24]. As indicated above for Rap1A retrogenes, also the hRap1B-retro may encode for a Rap1 protein with elevated GTP content because of a mutation at position twelve (G12R), but no functional characterization of the encoded protein was so far undertaken.

### Genomic localization and origin of Rap1A and Rap1B retrogenes

**Figure 3:**
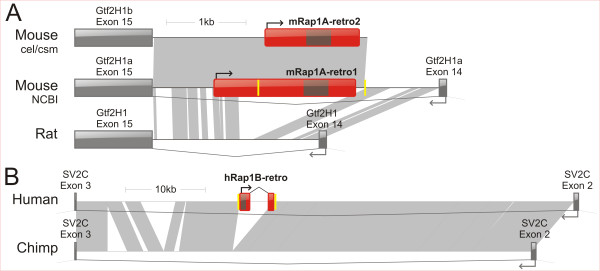
**Comparison of mRap1A-retro1, mRap1A-retro2 and hRap1B-retro insertion loci**. **A**. Existence of 2 genomic loci containing mRap1A-retro1 (Accession: EU359270) and mRap1A-retro2 (Accession: EU359271) is supported by (1) the NCBI chr.7 contig (NCBI, Accession: 20828562), (2) homologous sequences of the cellera assembly chr.7 contig (cel, Accession: 82906099) and the RZPD cosmid (csm, German Science Centre for Genome Research cosmid: MPMGc121C10270Q2) and the corresponding cDNAs of Gtf2H1a (Accession: 31982312) and Gtf2H1b (Accession: 2582796). As we resolved, by sequencing, the whole 5' UTR region of mRap1A-retro1, it is depicted as a longer message as compared to mRap1A-retro2. For alignments see [Additional file 2: Supplemental Figure S2]. Bottom: Rap1A retrogenes are absent from the orthologous loci in the rat genome. **B**. Based on the human-chimp genome comparisons we identified a sequence of the insertion/cleavage site: 5'-ctcaatcctaTTTT/Atgaaatccat-3' on the chr. 5 in the chimp genome. hRap1B-retro (GIs: 50474682, 50475494, 50470789, 50498405, 50492447) is located on the chr. 5 (Accession: 51465008). Red-colored boxes: transcripts of retrogenes. Dark-colored boxes within the retrogenes transcripts designate ORFs. Arrows mark a transcription direction. Yellow-colored boxes mark TSDs. Gray blocks mark regions of homology with an e-value < 10E-5.

BLAST searches with the mRap1A-retro1 cDNA sequence against the mouse genome sequence (NCBI 35) revealed that the gene is located on the antisense strand of the 14^th ^intron of general transcription factor II H, polypeptide 1 (Gtf2 h1) gene on chromosome 7 (see Figure 3A). The mRap1A-retro1 cDNA sequence mapped to a single exon - a typical feature of retrogenes. A sequence identical to that of mRap1A-retro2 was found on an RZPD cosmid (German Science Centre for Genome Research: MPMGc121C10270Q2) that overlapped with a Celera contig, also derived from chromosome 7 and containing exons of Gtf2 h1 (see Figure 3), [Additional file 2: Supplemental Figure S2-alignment]. (In contrast to the RZPD cosmid, the latter sequence contained a single point mutation resulting in a frame shift mutation in mRap1A-retro2, which may represent a sequencing error). Importantly, the Gtf2 h1 sequences flanking mRap1A-retro2 were not identical to those flanking mRap1A-retro1 and appeared to represent a second copy of the Gtf2 h1 gene. Both Gtf2 h1 genes appear to be transcriptionally active as is evident from two different cDNAs having intact ORFs and corresponding to Gtf2 h1a, Gtf2 h1b (Accession: 31982312 and Accession: 2582796). Importantly, mRap1A-retro1 and -retro2 retrogenes were not present in other mammalian genomes (i.e. Rap1A retrogenes were absent from the rat genome, but the Gtf2 h1 sequences flanking Rap1A retrogenes were highly homologous between mouse and rat, Figure 3A). Likewise, the duplicated Gtf2 h1 loci were only found in the mouse genome.

Moreover, phylogenetic analysis revealed that the group of the two Rap1A retrogenes and the mouse Rap1A gene form a monophyletic group in the tree with other mammalian Rap1A genes (with bootstrap support of 94%, see [Additional file 3: Supplemental Figure S3]). Thus, Rap1A retrogenes likely split off the mouse lineage. Together, this strongly implies that an event of insertion of a Rap1A retrogene into the 14th intron of Gtf2 h1 was followed by a duplication event and that the both events date back to times after rodent speciation. After the duplication, both Rap1A retrogenes diverged by non-synonymous mutations (i.e. mutations leading to amino acid changes) in their ORF regions (see the next subchapter).

We resolved the location of the hRap1B-retro retrogene to the region within the antisense strand of the 2^nd ^intron of SV2C (synaptic vesicle protein 2 isoform C) on chromosome 5 (Figure 3B). This Rap1B retrogene was only identified in the human genome sequence (i.e. was absent from the chimp genome), as revealed by genomic blast searches (see Methods).

Interestingly, hRap1B-retro's mRNA sequence maps to 2 exons that are separated by an intron, which was created by L1-mediated insertion of the SVA element into a preferred target site sequence (TTTT/AA) [25] present in human Rap1B mRNA (at 1304 nt, Accession: 58219793). We could reconstruct the insertion site for the human Rap1B retrogene based on the comparison with the chimp genome (Figure 3). The 2^nd ^intron of SV2C gene in the chimp genome bears the insertion site 5'-TTTT/A-3', typical of the site recognized by L1's endonuclease. Presence of target site duplications (TSDs) flanking Rap1A and Rap1B retrogenes provides further evidence for involvement of the LINE-1 retrotransposition machinery (Figure 3). Phylogenetic analysis established that hRap1-retro and the Rap1B in human and chimp form a monophyletic group (bootstrap support of 95%, see [Additional file 3: Supplemental Figure S3]). This implies that hRap1B-retro arose after divergence of macaca from the ancestor of chimp and human.

### Functional evolution scenario

**Figure 4:**
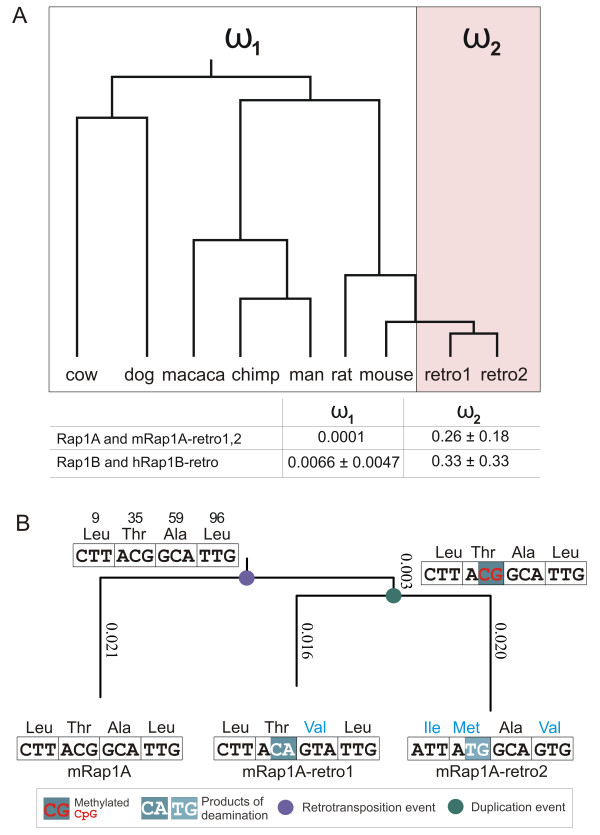
**Codon evolution model of Rap1 retrogenes**. **A**. The phylogenetic tree depicts the evolution of mammalian Rap1A orthologs and the two mouse Rap1A retrogenes. To check whether the two retrogenes are functional, we compute ω-ratios of non-synonymous to synonymous substitutions with the clades model of PAML. We assume one ω_1_-ratio for the left part of the tree whereas the evolution of recently emerged retrogenes is modelled by an individual ω_2_-value. Table shows the estimated ω-values and their standard deviations as computed by PAML. The amino acid sequences of mammalian Rap1A orthologs are fully conserved and the ML estimate ω_1 _= 0.0001 is too close to zero for PAML to compute a standard deviation. **B**. The evolution of the two mouse Rap1A retrogenes involved a retrotransposition event and a subsequent duplication event. Vertical edge lengths in the tree dendrogram were computed by the PAML software and scale with the expected number of neutral synonymous substitutions under the 2-clades codon evolution model. Leaves and internal nodes are annotated with the four codons at positions 9, 35, 59, 96, where non-synonymous subsitutions occured. Codons at internal nodes were reconstructed by applying the Maximum Parsimony principle to single nucleotide substitutions. It can be hypothesized, that non-synonymous (T35 M) and synonymous (T35T) mutations at codon 35 observed in mRap1A-retro1 and mRap1A-retro2, respectively, result from deaminations of methylated CpG dinucleotide. Non-synonymous mutations are highlighted in cyan.

Could Yang's codon evolution model provide evidence for the functionality of the retrogenes? If the retrogenes are functional, we expect that there are selective constraints on amino acid changing substitutions. As a consequence, the ratio ω of non-synonymous to synonymous nucleotide substitution rates should be significantly smaller than one for the retrogenes. To check that, we analyzed multiple alignments of the Rap1A and Rap1B genes with orthologs in human, chimp, macaca, dog, cow, rat, and mouse. All computational analysis described below were performed by means of the codeml program of the PAML software package (v. 3.15) [26]. Codeml assumes a time-continuous Markov process of codon evolution [27] and ω-ratios were computed as Maximum-Likelihood-Estimates that maximize the probability to observe the multiple alignment under the assumptions specified below as outcome of the Markov process. We also computed standard deviations of the ω-ratios by means of codeml. The assumed stationary distribution of codons was set to the one that reflects the empirical nucleotide frequencies at the three codon positions in the multiple alignment. Further, since the gene tree did not fully resolve relations between the Rap1 genes [Additional file 3: Supplemental Figure S3], we assumed that orthologs in the alignment have evolved along the branches of the established species phylogeny [28] (see Methods). Because the mouse retrogenes are most similar to the mouse ortholog, as supported by the well-resolved parts of the gene tree [Additional file 3: Supplemental Figure S3], we assume the retrogenes split off the mouse lineage. For example, Figure 4A shows the phylogenetic tree with orthologs of Rap1A and the two retrogenes in mouse. The right hand part of the tree depicts the evolution of the two retrogenes in mouse that emerged by a retrotransposition and a duplication event. We assume the so called clades model (of PAML), i.e. the codon evolution model where the mammalian orthologs have evolved in accordance to one ω-ratio ω_1_, whereas the evolution of the retrogenes was governed by a different ω-ratio ω_2_. The multiple alignment of Rap1B genes and the respective human retrogene was modelled analogously under the clades-model. Amino acid sequences of mammalian Rap1A and Rap1B proteins are highly conserved, which is reflected in the small ω_1_-values indicating strong negative selection on Rap1A and Rap1B genes. We find that, ω_2_-values are much larger than the ω_1_-values.

We tested whether it is appropriate to assume two different ω-ratios in the tree within the clades model by computing the likelihood under the simpler M0-model of codeml and performing a Likelihood Ratio Test (LRT). The simpler M0-model, which assumes only one ω-ratio for the whole tree, is a special case of the clades model. Because the M0 model is nested in the clades model, the likelihood under the clades model is by definition larger or equal than the likelihood under the M0-model for every alignment. The test statistic Δ of the LRT is two times the difference in log likelihoods, Δ = 2 ln L(clades-model) - ln L(M0). The null assumption H_0 _of the LRT corresponds to the assumption that sequences in the multiple alignment had evolved according to the simpler M0-model. Then, under the null assumption H_0_, the expected distribution of Δ is a χ^2 ^distribution with one degree of freedom. The LRT reveals for both alignments that the clades model with two ω-ratios performs significantly better than the M0-model of codeml, computed p-values were p_rap1A _< 10^-6 ^and p_rap1B _= 0.0013.

The fact that ω-ratios are larger for retrogenes suggests that the selective pressure on the replacement of amino acids was relaxed during the evolution of the retrogenes. However, ω_2 _estimates are at least two standard deviations smaller than one for both, for the alignment of Rap1A genes as well as for the alignment of Rap1B genes. Thus, synonymous substitutions, which are assumed to be neutral in Yang's codon substitution model, occurred significantly more often than non-synonymous substitutions and we argue that the evolution of retrogenes was subject to moderate selective constraints.

Interestingly, Lynch et al. [28] compared measures of ω in paralogous genes of several eukaryotic genomes. The authors demonstrated that ω systematically decreases in time and concluded that "early in their evolutionary history, duplicated genes tend to be under moderate selective constraints" where the range for ω lies between values of 0.37 and 0.46. We thus argue, that the values of ω_2_(Rap1A) = 0.26 and ω_2_(Rap1B) = 0.33 indicate that the evolution of mRap1A-retro1, -retro2 genes and hRap1B-retro is constrained by a regime of at least "moderate selection".

The tree dendrogram of Figure 4B shows the evolution of the two mouse Rap1A retrogenes after a retrotransposition event and a subsequent duplication event. If we assume the expected number of neutral synonymous substitutions under the 2-clades codon evolution model (described above) to be proportional to the elapse of time, then it can be speculated that the duplication event occurred only shortly after the retrotransposition event. Moreover, we found evidence for a scenario according to which the mutations at codon 35 resulting in synonymous and non-synonymous (T35 M) substitutions in mRap1A-retro1 and mRap1A-retro2, respectively, were generated by deamination of methylated CpG (Figure 4b).

We further employed structural analysis and functional assays to uncover evidence for the molecular basis of evolutionary pressure to conserve these genes.

### Structure-related functional analysis

In order to functionally characterize the Rap1 retrogene encoded proteins, we first tested for their ability to bind guanine nucleotides. mRap1A-retro1 and hRap1B-retro contain mutations in 59^th ^(in G3 motif) and 12^th ^(in G1 motif) amino acid, respectively, that are involved in the control of interactions with the GTP ligand (Figure 1B). Since mutations at position 12 and 59 are transforming mutations in Ras [29-31], it is expected that human Rap1B-retro and mouse mRap1A-retro1 are more GTP-bound than wild type Rap1.

**Figure 5:**
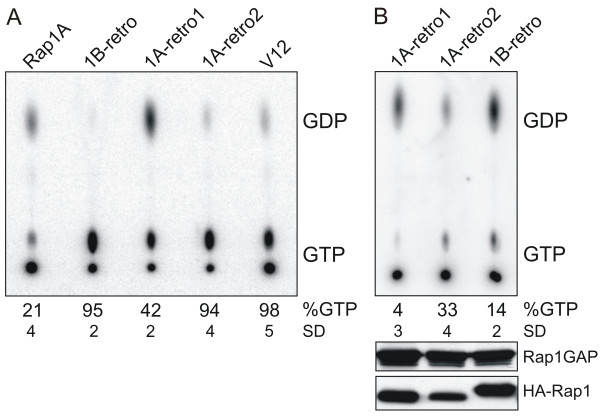
**Determination of the GTP/GDP ratio bound to mRap1A-retro1, -retro2 and hRap1B-retro**. **A**. Comparison of GTP binding between Rap1 retrogenes and Rap1V12. Cos-7 cells were transfected with 2 μg different Rap1 constructs as indicated, starved overnight, labelled with [32P] orthophosphate for 5 h. HA-Rap1 was immunoprecipitated with the 12CA5 anti-HA monoclonal antibody, and bound nucleotides were separated using thin layer chromatography, followed by PhosphorImager detection. SD, standard deviation. **B**. Rap retrogenes are sensitive to Rap1Gap. Cos7 cells were transfected with HA-Rap1Gap in combination with mRap1A-retro1, mRap1A-retro2 and hRap1B-retro. GDP and GTP, bound to transfected Rap1, was measured as indicated above. The bottom panels indicate the expression of HA-Rap1Gap and HA-Rap as determined by Western blotting using anti-HA as a probe.

Cos-7 cells were transfected with HA-tagged versions of wild type Rap1A, human Rap1B-retro, mouse mRap1A-retro1, mouse mRap1A-retro2 or Rap1A-V12 and labeled with [^32^P]. HA-tagged proteins were isolated by immuno-precipitation and their GTP/GDP content was determined by thin layer chromatography (Figure 5A). As we and others reported previously, the percentage of GTP bound to wild type Rap1A is around twenty percent, while Rap1A-V12 is highly GTP-bound [13,32]. Indeed, more than ninety percent of human Rap1B-retro and about forty percent of mRap1A-retro1 was GTP-bound. mRap1A-retro2 was also almost exclusively GTP-bound. The latter is most likely explained by the mutation in the T35 located in the switch I region corresponding to the general recognition motif in the effector domain (Figure 1). Importantly, T35 is conserved in Ras-like proteins [33]. Inspection of the crystal structure of the complex between the Rap1A and the Ras-binding domain of c-Raf1 shows that T35 helps in coordinating the gamma-phosphate of GTP and the attacking water molecule required for hydrolysis (Figure 1B).

Previously, it has been reported that V12 mutations in Rap1 are still sensitive to Rap1GAP [34].

GAPs (GTPase activating proteins) are proteins that mediate negative regulation of Ras-like GTPases by catalyzing the hydrolysis of GTP. Co-transfection of Rap1GAP with any of the retrogenes caused a strong decrease in the GTP/GDP ratio. However, this decrease was clearly less pronounced for mRap1A-retro2 (Figure 5C) underlining the importance of T35 for GTP hydrolysis but also its involvement in control of interactions with target proteins. It was shown that mutations in T35 residue perturb a dynamic behavior of the switch I region [35] and this can affect interactions with effector proteins but also regulatory factors like GAP which bind to this region [36].

**Figure 6:**
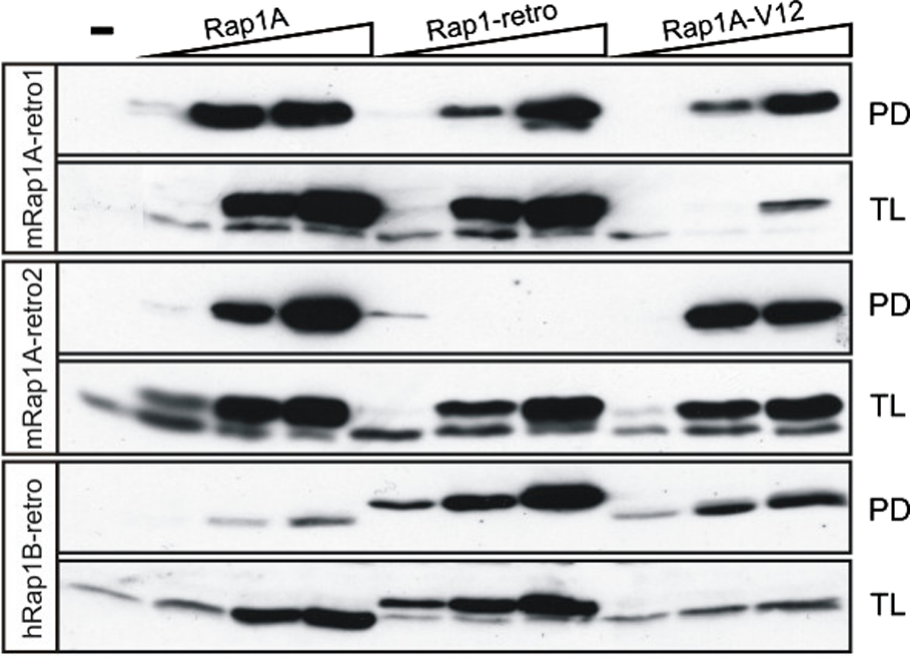
**Analysis of activation of mRap1A-retro1, -retro2 and hRap1B-retro**. Cos7 cells were transfected with empty vector, 0.1, 0.5 or 2 μg Rap1A (Rap1A), 0.1, 0.5 or 2 μg Rap1 retrogenes or 0.1, 0.5 or 2 μg Rap1A-V12. After 48 hours cells were lysed, and Rap1-GTP was isolated using GST-RalGDS-RBD and detected by using Western blotting with an anti-HA antibody, indicated as PD: pull down. The expression of HA-Rap1 was determined by Westernblotting using anti-HA as a probe, indicated as TL: total lysate.

The result above indicates that the conformations of the proteins encoded by retrogenes resemble that of active GTP-bound Rap1. GTP-bound Rap1 and Rap1-V12 are known to strongly interact with the Ras-binding domain (RBD) of RalGDS [37]. Therefore, we compared the binding of the retrogene-encoded isoforms to GST-RBD with that of Rap1A-V12 using a pull down assay (Figure 6). The binding of hRap1B-retro and mRap1A-retro1 to RalGDS RBD was comparable to that of Rap1A-V12. In contrast, mouse mRap1A-retro2 did not significantly bind to this RBD. As discussed above, this is most likely caused by the T35 M mutation in the effector domain region that mediates the interaction with the RBD (see Figure 1B).

One of the best-documented functions of Rap1 is to enhance integrin-dependent adhesion to extra-cellular matrices [38]. We assayed the ability of retrogene isoforms to increase adhesion of HB6 cells (Jurkat T cells expressing Epac1).

**Figure 7:**
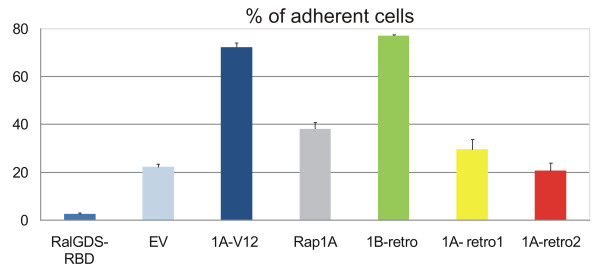
**Analysis of the effect of mRap1A-retro1, -retro2 and hRap1B-retro on cell adhesion**. Jurkat cells stably expressing Epac (HB6 cells) were co-transfected with TK luciferase reporter plasmid and either empty vector (EV) or Rap1 constructs as indicated. After 42 h cells were replated and seeded in triplicate wells coated with fibronectin and after 1 h the percentage of adherent cells was measured. Results are representative of three independent experiments. The expression of HA-Rap and HA-RalGDS-RBD was determined by Western blotting using anti-HA as a probe (data not shown).

Normally, these cells remain in suspension when plated on fibronectin-coated surfaces, but when activated Rap1A is introduced into these cells they adhere in a VLA-4 (α_4_β_1_) dependent manner. We electroporated expression vectors for the various Rap1 isoforms into HB6 cells and the next day allowed them to adhere to fibronectin-coated dishes (Methods). Both human Rap1A-V12 and human Rap1B-retro clearly increased adhesion (Figure 7). As reported previously, expression of the RBD of RalGDS decreased adhesion by preventing endogenous Rap1 effectors to interact with Rap1. Strikingly, expression of mouse retrogenes did not affect adhesion positively or negatively. From this result we conclude that whereas human Rap1B-retro has dominant-active properties in terms of regulation of integrin-mediated adhesion, the mouse retrogenes are inactive in this respect.

**Figure 8:**
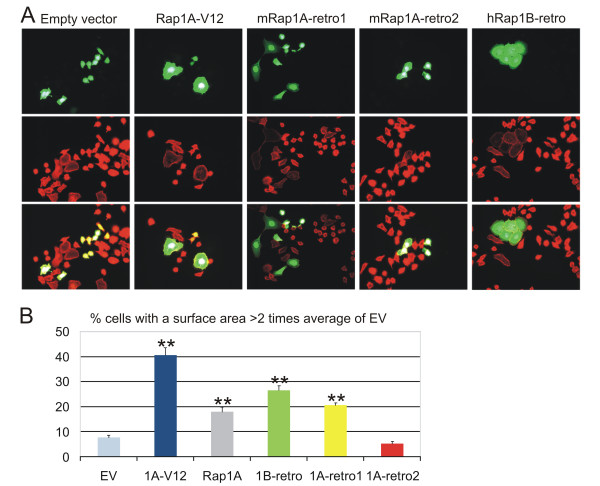
**Analysis of the effect of mRap1A-retro1, -retro2 and hRa1pB-retro on cell spreading**. **A**. A431 cells were co-transfected with histon2B-GFP and Rap1 constructs as indicated. Cells were grown overnight in tissue culture wells and then photographed. GFP-positive cells were visualized by phalloidin (red) staining. **B**. For cell spreading, first we determined the average surface of >100 non-transfected cells using the ImageJ program. Next, the surface of at least 90 transfected cells (selected on the basis of the presence of GFP-H2B) from two independent experiments was measured. Cells with a surface area of more than twice that of untransfected cells were scored as spread. ** indicates a p-value < 0.001 (Chi-test).

Cell spreading is a second function of Rap1, which is independent of the control of integrin-mediated adhesion [39]. To test for this function, retrogenes were co-transfected with histon2B-GFP into A431 cells, grown on glass coverslips, followed by staining with the anti-HA antibody 12CA5 or phalloidin. First we confirmed that virtually all cells expressing GFP also co-expressed HA-tagged Rap1 (data not shown). Next, we used the phalloidin staining to measure the surface area of GFP-positive cells. Untransfected and empty vector-transfected cells mostly have a round morphology under these conditions. Expression of HA-Rap1V12, HA-hRap1B-retro and HA-mRap1A-retro1 induced a clear increase in the fraction of spread cells (Figure 8A and 8B). In contrast, HA-Rap1A-retro2 did not significantly alter cell spreading.

Together these results demonstrate that the proteins encoded by the three Rap1 retrogenes differ in their biological activities.

## Discussion

Reverse transcription of mRNAs followed by insertion into the genome is an event, which in germ cells can yield additional copies of genes for future generations. In most cases they will develop into transcriptionally silent pseudogenes, which harbor mutations in their coding regions, preventing the production of functional proteins. However, if retrotransposition yields processed genes that have a distinct tempero-spatial expression pattern and/or have acquired neomorphic functions as a consequence of mutation, their presence may be advantageous to the organism and expression may be maintained [40].

Indeed, a number of reports have recently appeared, suggesting that expression of processed genes (termed retrogenes) might be more common than previously anticipated. For example, the DPPA3 gene, which has been used as a marker for pluripotency, was found to have a number of related retrogenes, of which at least one was found to be expressed in adult tissues [41]. Expressed retrogenes are not simple evolutionary relics, but can obtain essential functions as was shown for the Utp14B protein. Mutation of this autosomal gene results in juvenile spermatogonial depletion [42].

In the course of analysis of our Rap1A knockout mice, we isolated cDNAs from various tissues, corresponding to two distinct Rap1A retrogenes. The existence of Rap1-like retrogenes appears not to be unique to mice. Recently, we and others reported the existence of hRap1B-retro retrogene in human, whose expression might be linked to development of myelodysplastic syndromes and other types of leukemia [23,24]. Genomic comparisons indicated that the Rap1A and Rap1B retrogenes are species-specific and that the Rap1A retrogenes are paralogs. Interestingly, the recently emerged Rap1 retrogenes acquired limited amino acid-changing substitutions and our statistical analysis favors a scenario whereby they evolved under selective constraints on coding sequence. In this respect, hRap1B-retro (G12R, K42E) and mRap1A-retro1 (A59V) were of special interest since they had substitutions at positions 12 and 59 (Figure 1), which resembled those found in constitutively active oncogenic Ras proteins. Both retrogene-encoded isoforms had an increased GTP/GDP binding ratio and interacted strongly with the Ras-binding domain of RalGDS in pull-down assays. Strikingly, only hRap1B-retro increased integrin-mediated cell adhesion, whereas mRap1A-retro1 was practically inactive in this assay. Cell spreading on the other hand was increased upon over-expression of both hRap1B-retro and mRap1-retro1. These results are reminiscent of those obtained with various Rap1 mutants, generated via site directed mutagenesis [43]. In that study, Rap1A G12V appeared to be more powerful in suppressing growth of tumorigenic HT1080 cells than wild type Rap1, but its capacity to induce a flat revertant phenotype of in Ras-transformed DT cells was not strongly increased. In contrast, a Rap1A A59T mutant showed opposite characteristics with a mild effect on HT1080 proliferation but a clearly enhanced effect in the flat revertant assay. Together, these results indicate that the proteins encoded by the Rap1 retrogenes have different biological activities, and uncouple cell adhesion and cell spreading, possibly by activating distinct (sets of) Rap1 effectors. Indeed, Rap1 effectors have been described, which preferentially increase cell spreading like VAV [39] or both cell spreading and cell-matrix interaction like RIAM [44]. It will be interesting to see if the Rap1 retrogene products indeed selectively interact with such effectors. The fact that Rap1 retrogene encoded proteins are in a GTP-bound state suggest that retrogene expression would allow for activation of Rap1-dependent processes in a stimulus-independent way. This phenomenon is well known in the case of Ras, where somatic mutations result in oncogenic proteins, which stimulate the ERK and PI-3K pathways in the absence of growth factors. Likewise, expression of Rap1 retrogenes may activate the above-mentioned effectors VAV or RIAM or any of the other Rap1-effectors that include diverse proteins like phospholipase Cε or the scaffold protein AF6 (reviewed in [45]). The precise mode by which retrogene encoded Rap1 isoforms interact with known Rap1 effectors or perhaps additional proteins will determine the physiological consequences. These may include effects on cell adhesion, important for neural pathfinding and immune surveillance or cell-cell adhesion, contributing e.g. to vascular permeability.

## Conclusions

Collectively, the data above strongly indicate that mouse mRap1A-retro1 and human hRap1B-retro retrogenes represent recent examples of functional evolution. However, it has to be taken into consideration that not all of the described retrogenes will be selectively maintained and neo-functionalized. Indeed, we have not been able to detect a biological effect of mRap1A-retro2 over-expression. This may lend support for the scenario where mRap1A-retro2 is unlikely to escape non-functionalization.

Although it is hard to rule out that the other two retrogenes are on their way to non-functionalization, the fact that mRap1A-retro1 solely stimulates cell spreading but not adhesion, points to a unique property of this isoform. In addition, the fact that it like hRap1B-retro encodes for constitutively active protein, indicates that it can function largely independently of GEFs. Although one could argue that our current study does not reveal truly novel functions to warrant the term neo-functionalization, future studies that should try to identify downstream effectors for e.g. mRap1A-retro2 should shed further light on this issue.

It is of note that the Ras-like protein family contains at least two other members of retrogenic origin, a H-Ras retrogene named Eras, and a representative of Rap subfamily, Rap2B. Interestingly, mouse and human ERas, acquired mutations at positions 12, 59, 62 that render constitutively active proteins [46]. ERas is absent from genomes of fish and amphibians and appears to be exclusively present in mammalian genomes. Its primary function was linked to its highly specific domain of expression in mouse ES cells [46], yet expression of ERas was not detected in human ES cells [47]. On the other hand, the Rap2B retrogene, originally discovered in human platelets [48], is vastly conserved in vertebrate genomes. By contrast, Rap1 retrogenes are products of recent evolutionary events that created species-specific isoforms with differential signaling capacity. Although their physiological significance remains to be established, the fact that all of them are GTP-loaded, yet have distinct signaling capacities, shows how limited amino acid substitutions may turn recently emerged retrogenes into functionally novel genetic entities.

## Methods

### Sequences of Rap1 orthologs

The sequences of Rap1A and Rap1B orthologs were extracted using human and mouse Rap1A/B sequences (NM_145541, NM_015646) as queries in tblastx searches against the chimp, macaca, rat, dog and cow genomes.

### Genomic comparisons

In order to identify orthologous loci of mouse Rap1A and human Rap1B retrogenes in the rat and in the chimp genomes, respectively, we utilized nucleotide and protein blast searches. We checked for presence/absence of Rap1B and Rap1A retrogenes in the sequences of human, chimp, orangutan, rhesus, marmoset, mouse, rat, guinea pig, cat, dog, horse, cow, opossum and platypus genomes (as made available at the UCSC genome browser). The multiple alignments reported in the [Additional file 2: Supplemental Figure S2] and in the [Additional file 3: Supplemental Figure S3] were computed with ClustalX and visually inspected afterwards.

### Phylogenetic analysis

Given the alignment of all Rap1 genes, we wanted to infer a gene tree according to which the Rap1 genes putatively evolved. We used jModelTest (version 0.1.1) to identify the nucleotide substitution model that best fits the alignment [49] and PhyML [50] to infer possible gene trees. The "HKY85 + I" nucleotide substitution model (which parameterizes substitution rates with a parameter for the transition-transversion ratio, accounts for different nucleotide frequencies, and assumes that a fraction of sites is invariable) was always selected as the best-fit model according to the Akaike Information Criterion (with and without second order correction) and the Bayes Information Criterion.

We then ran PhyML with "HKY85 + I" as nucleotide substitution model on the alignment as well on 100 bootstrap replicates of the alignment. The resulting tree with annotated bootstrap values is shown in [Additional file 3: Supplemental Figure S3].

The ML tree does not reflect the accepted species phylogeny. However, the Shimodaira-Hasegawa (SH) [51] test to compare different assumptions about the tree revealed that the maximal likelihood of the tree computed with PhyML is not significantly different from the likelihood under the tree that reflects the species phylogeny (shown in Figure 4).

Compounding both, the SH test and weak bootstrap support values at basal branches, we concluded that the alignment is too conserved to carry enough signal to resolve basal branching patterns. We therefore assumed that orthologs in the alignment have evolved along the branches of the established species phylogeny (Figure 4) and confirmed that the computation of ω values is not sensitive to different basal branching patterns. For example we considered the topology ((rodents, (cow, dog)), primates) and compared results to the ones under ((rodents, primates), (cow, dog)) which represents the established species phylogeny. The ω values shown in Table in Figure 4A are the same under the two alternative tree topologies.

### RNA isolation and reverse transcription

RNA was isolated using standard protocols and deprived of DNA using the DNAseI kit from Promega Corporation according to the manufacturer's recommendations.

cDNA was made using the SuperscriptTM II Reverse Transcripatase kit from Invitrogen according to the manufacturer's protocol. For each RNA sample a control reaction was prepared w/o RT polymerase to check whether Dnase I digestion had been successful.

### RT- PCR reaction and cloning of retrogenes

For PCR reactions, the Taq PCR core kit fom Qiagene was used. 2-4 μl of RT reaction was mixed with PCR buffer, dNTPs, water, Taq polymerase and 10 pmol of 5' and 3' specific primers. The following PCR conditions were used: 96°C, 2 min. 25-35 cycles: 96°C-20 s, Tm-50°C -20 s, 72°C-1 min.: 72°C, 10 min. Using the following primer set we amplified a 1186 bp fragment of Rap1A as well as mRap1A-retro1 and mRap1A-retro2 genes: 5'-GCGGGATTGTCAATATTTAAAC-3' and 5'-GCCATAGAAATCAGTTATCCC-3'. This primer set amplifies the whole ORF and a part of the 3'UTR. To specifically amplify Rap1A retrogenes from Rap1A -/- tissues one primer was set in the targeted second exon: 5'-TCAGGAGGCGTGGGGAAG-3' and the other in the 3' UTR: 5'-GCCATAGAAATCAGTTATCCC-3'. PCR products from multiple tissues (i.e. thymus, liver, spleen, testis) were cloned into vector from TOPO TA cloning kit (Invitrogen) and then sequenced using standard primers recommended by manufacturer.

For the expression and activity studies both retrogenes were amplified using Pfu polymerase and primers compatible for cloning full length proteins into pMT2HA.

### Gene Racer reaction - full length RNA ligase-mediated rapid amplification of 5' cDNA ends (RLM-RACE PCR)

The Gene Racer kit (Invitrogen) was used according to the manufacturer's recommendation. Briefly, RNA was dephosphorylated to eliminate truncated mRNA and non-mRNA from subsequent ligation with the GeneRacer RNA Oligo. Next, the cap structure from intact, full-length mRNA was removed using Tap enzyme. This treatment generates a 5'phosphate required for ligation to the "GeneRacer RNA Oligo". Subsequently, ligation of RNA Oligo to decapped mRNA was performed utilizing T4 RNA ligase. Then, cDNA was produced on this RNA using random primers. Hereafter, PCR was performed in order to obtain 5' ends. For this purpose, the first-strand cDNA was amplified using "GeneRacer 5' Primer" (homologous to the GeneRacer RNA Oligo): 5'-CGACTGGAGCACGAGGACACTGA-3' and a reverse gene-specific primer located in the targeted second exon: 5'-CTTCCCCACGCCTCCTGAACCAA-3'. Only mRNA that ligated to ''GeneRacer RNA Oligo" and is completely reverse transcribed will be amplified. This PCR was prepared the standard way using Taq polymerase from Quiagen. Subsequently, this PCR reaction was further used for nested PCR. Here, "5'GeneRacer Nest primer": 5'-GGACACTGACATGGACTGAAGGAGTA-3' and reverse nested gene specific primer located in the targeted second exon: 5'-CTTCCCCACGCCTCCTGAACCAA-3' were used. The products obtained as single dominant bands were then cloned into pCR4-TOPO vector (TOPO TA cloning kit Invitrogen) and sequenced. The sequence derived from wild type material, was a 5' sequence of mRap1A mRNA. The PCR product from knockout material was corresponding to a 5' UTR of mRap1A-retro1. The 5'UTR region of mRap1A-retro1 is longer (by ~550 nt) than the 5'UTR of mRap1A and different from the 5' UTR of mRap1A (as also evident from the genomic sequence of mRap1A-retro1).

### Sequencing of Cosmid DNA harboring mRap1A-retro2

Cosmid MPMGc121C10270Q2, obtained from RZPD (German Science Centre for Genome Research) was identified during the screen for the Rap1A gene. We sequenced 1741 nt of this cosmid harboring mRap1A-retro2, using the following oligos:

5'-GCGGGATTGTCAATATTTAAAC-3'; 5'-CTTCCCCACGCCTCCTGAACCAA-3'; 5'-CACCAGTGGAAAAGAAGAAGCC-3' and 5'-GGCACAGTTACACCACTGTCTTG-3'.

### Cell culture

Cos-7 cells were grown in Dulbecco's modified Eagles medium (DMEM) containing 10% fetal calf serum (FCS) and serum-starved in DMEM at 1.5% FCS. A431 cells expressing human p120 short interfering RNA (siRNA) was described previously [52]. Cells were cultured in DMEM containing L-glutamine (Hyclone), 10% fetal bovine serum (FBS) (Hyclone), and 1% penicillin/streptomycin (Gibco/Invitrogen).

### Cloning of hRap1B-retro into pMT2HA vector

The fragment containing the ORF of hRap1Bretro was amplified from human genomic DNA using primers: 5'-ATGTCGACCATGCGTGAGTATAAGCTAGTCGT-3' and 5'-ATGCGGCCGCAGACCTGGCTCAGAGCTACA-3'. We used Advantage polymerase from Clontech with PCR conditions: 1 cycle: 95°C 1 min./35 cycles: 95°C 15 sec, 68°C 1 min/1 cycle: 68°C 10 min. The PCR product was cloned into vector from TOPO TA cloning kit (Invitrogen) and then re-cloned into SalI and NotI of pMT2HA vector.

### Activity measurements for Rap1 and related proteins

Determination of the GDP/GTP ratio using [^32^P]-labeling of cells or using a non-radio active pull-down assay were done as described in Zwartkruis et al. 1998 [13]. Haemaglutamin (HA)-tagged Rap1, Rap1V12 (HA-RapV12), Rap1GAP (HA-RapGAP I) have previously been described [13].

### Cell spreading assay

To measure cell spreading, A431 p120 RNAi cells [52] were plated on glass cover slips. The next day cells were transfected with either empty vector or pMT2HA vectors expressing the various Rap1 cDNAs and histon2B::GFP using Fugene. After 24 hours, cells were fixed and stained either for the HA-tag to confirm that GFP positive cells also expressed Rap1 protein or with TRITC phalloidin. Cell surface area was measured using the ImageJ program version 1.36b (NIH).

### Adhesion Assay

For adhesion assays, transiently transfected Jurkat cells, serum starved overnight, were harvested, washed, and suspended in TSM buffer (20 mM Tris-HCl, pH 8.0, 150 mM NaCl, 1 mM CaCl_2_, 2 mM MgCl_2_) at a concentration of 5 × 10^5 ^cells/ml. 24-Well Nunc Maxisorp plates (Corning, NY, USA) were coated with fibronectin (5 μg/ml) overnight at 4°C, washed, and blocked for 1 hour at 37°C with 1% bovine serum albumin (BSA), TSM. After washing, 200 μl of TSM was added per well with or without the indicated stimuli. Subsequently, 200 μl cell suspension was added per well. Cells were allowed to adhere for 1 hour at 37°C and non-adherent cells were removed with warmed 0.5% BSA, TSM. Adherent cells were lysed and subjected to a luciferase assay as described previously [53]. Expression of transfected constructs was confirmed by immunoblotting of total cell lysates. Adherent cells were calculated, and the cell numbers were corrected for transfection efficiency and non-specific effects of constructs by measuring luciferase activity of total input cells ((counts in cells bound/counts in total input cells) × 100%).

## Authors' contributions

TZ co-initiated and coordinated the project, co-identified the retrogenes, co-performed the statistical and genomic analysis and co-wrote the manuscript, MD co-identified and cloned the retrogenes, ZZ performed adhesion assays, TPaluch cloned expression vectors for the retrogenes and did the pull down experiments, HL co-performed the statistical analysis and co-wrote corresponding part of the manuscript, TPenzkofer co-performed the genomic analysis, JS participated in the initiation and coordination of the project, FZ co-initiated and coordinated the project, did the hot labeling assays and cell spreading assays and co-wrote the manuscript. All authors read and approved the final manuscript.

## Supplementary Material

Additional file 1 **Supplemental Figure S1**. RT- PCR for Rap1A-retro1 and Rap1A-retro2 retrogenes in WT organs. Click here for file

Additional file 2 **Supplemental Figure S2**. Alignment between cDNAs of Rap1A-retro1, Rap1A-retro2 and the corresponding genomic regions. Click here for file

Additional file 3 **Supplemental Figure S3**. The gene tree for the Rap1 genes. Click here for file
