# Supplementary material for: Retrotransposition and mutation events yield Rap1 GTPases with differential signalling capacity
Source: BMC Evol Biol. 2010 Feb 19;10:55. doi: 10.1186/1471-2148-10-55 (PMC2831893; doi:10.1186/1471-2148-10-55)
Supplement: Additional file 1 — Supplemental Figure S1. RT- PCR for Rap1A-retro1 and Rap1A-retro2 retrogenes in WT organs. [file 1471-2148-10-55-S1.DOC]

**Supplementary Figure 1.** RT- PCR for Rap1A-retro1, -retro2 retrogenes in WT organs. Retrogene-specific primers were used (see Materials and methods). The first lane is a marker. **
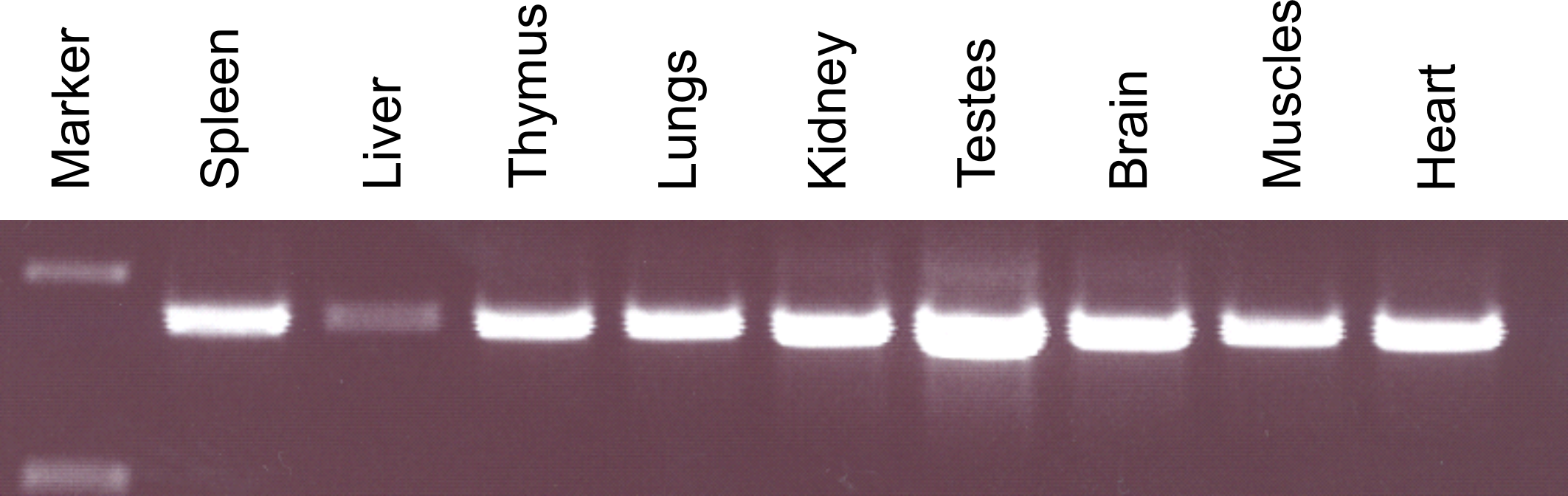
**
