# Supplementary material for: Retrotransposition and mutation events yield Rap1 GTPases with differential signalling capacity
Source: BMC Evol Biol. 2010 Feb 19;10:55. doi: 10.1186/1471-2148-10-55 (PMC2831893; doi:10.1186/1471-2148-10-55)
Supplement: Additional file 2 — Supplemental Figure S2. Alignment between cDNAs of Rap1A-retro1, Rap1A-retro2 and the corresponding genomic regions. [file 1471-2148-10-55-S2.DOC]

**Supplementary Figure 2.** Alignment between cDNAs of Rap1A-retro1 (Accession: EU359270), Rap1A-retro2 (Accession: EU359271), Rap1A (GI: 142365908) and the corresponding genomic regions on the chr. 7. NCBI, GI: 20828562; cel, GI: 82906099; csm: cosmid MPMGc121C10270Q2; Gtf2H1a (GI: 31982312) and Gtf2H1b (GI: 2582796).

10 20 30 40 50 60 70 80 90 100 110 120

....|....|....|....|....|....|....|....|....|....|....|....|....|....|....|....|....|....|....|....|....|....|....|....|

Rap1A-retro1 cDNA **~~~~~~~~~~~~~~~~~~~~~~~~~~~~~~~~~~~~~~~~~~~~~~~~~~~~~~~~~~~~~~~~~~~~~~~~~~~~~~~~~~~~~~~~~~~~~~~~~~~~~~~~~~~~~~~~~~~~~~~~**

Rap1A-retro1 NCBI **TTGGGGACTCAGAACACAGGCTTCAGTGCCACACTTATTTCCGTAGGAAACACAGGACTAGTGGAGTCTTCTGACAACTGTTTCCTGATGGCTGAGCAACAGCATCCCTCTGCCTTATTT**

Gtf2h1a **TTGGGGACTCAGAACACAGGCTTCAGTGCCACACTTATTTCCGTAGGAAACACAGGACTAGTGGAGTCTTCTGACAACTGTTTCCTGATGGCTGAGCAACAGCATCCCTCTGCCTTATTT**

Rap1A-retro2 cDNA **~~~~~~~~~~~~~~~~~~~~~~~~~~~~~~~~~~~~~~~~~~~~~~~~~~~~~~~~~~~~~~~~~~~~~~~~~~~~~~~~~~~~~~~~~~~~~~~~~~~~~~~~~~~~~~~~~~~~~~~~**

Rap1A-retro2 csm **~~~~~~~~~~~~~~~~~~~~~~~~~~~~~~~~~~~~~~~~~~~~~~~~~~~~~~~~~~~~~~~~~~~~~~~~~~~~~~~~~~~~~~~~~~~~~~~~~~~~~~~~~~~~~~~~~~~~~~~~**

Rap1A-retro2 cel **TTGGGGACTCAGAACACAGGCTTCAGTGCCACACTTATTTCCGTAGGAAACACAGGACTAGTGGAGTCTTCTGACAACTGTTTCCTGATGGCTGAGCAACAGCGTCCCTCTGCCTTATTT**

GtF2h1b **~~~~~~~~~~~~~~~~~~~~~~~~~~~~~~~~~~~~~~~~~~~~~~~~~~~~~~~~~~~~~~~~~~~~~~~~~~~~~~~~~~~~~~~~~~~~~~~~~~~~~~~~~~~~~~~~~~~~~~~~**

Rap1A cDNA **~~~~~~~~~~~~~~~~~~~~~~~~~~~~~~~~~~~~~~~~~~~~~~~~~~~~~~~~~~~~~~~~~~~~~~~~~~~~~~~~~~~~~~~~~~~~~~~~~~~~~~~~~~~~~~~~~~~~~~~~**

130 140 150 160 170 180 190 200 210 220 230 240

....|....|....|....|....|....|....|....|....|....|....|....|....|....|....|....|....|....|....|....|....|....|....|....|

Rap1A-retro1 cDNA **~~~~~~~~~~~~~~~~~~~~~~~~~~~~~~~~~~~~~~~~~~~~~~~~~~~~~~~~~~~~~~~~~~~~~~~~~~~~~~~~~~~~~~~~~~~~~~~~~~~~~~~~~~~~~~~~~~~~~~~~**

Rap1A-retro1 NCBI **AAAGCACTCTCTTTAACATGC~TGAAGTAAGGAACAACTAGAATTTAGTAACATTTGAGGATCCCAGTGTGGGTCTGAGGAAGGATTCTTAGGGCTACCCTGTGCCACAAACTTAGGGAA**

Gtf2h1a **AAAGCACTCTCTTTAACATGC~TGAAGTAAGGAACAACTAGAATTTAGTAACATTTGAGGATCCCAGTGTGGGTCTGAGGAAGGATTCTTAGGGCTACCCTGTGCCACAAACTTAGGGAA**

Rap1A-retro2 cDNA **~~~~~~~~~~~~~~~~~~~~~~~~~~~~~~~~~~~~~~~~~~~~~~~~~~~~~~~~~~~~~~~~~~~~~~~~~~~~~~~~~~~~~~~~~~~~~~~~~~~~~~~~~~~~~~~~~~~~~~~~**

Rap1A-retro2 csm **~~~~~~~~~~~~~~~~~~~~~~~~~~~~~~~~~~~~~~~~~~~~~~~~~~~~~~~~~~~~~~~~~~~~~~~~~~~~~~~~~~~~~~~~~~~~~~~~~~~~~~~~~~~~~~~~~~~~~~~~**

Rap1A-retro2 cel **AAAGCACTCTCTTTAACATGCGTGAAGTAAGGAACAACTAGAATTTAGTAACATTTGAGGATCCCAGTGTGGGTCTGAGGAAGGATTCTTAGGGCTACCCTGTGCCACAAACTTAGGGAA**

GtF2h1b **~~~~~~~~~~~~~~~~~~~~~~~~~~~~~~~~~~~~~~~~~~~~~~~~~~~~~~~~~~~~~~~~~~~~~~~~~~~~~~~~~~~~~~~~~~~~~~~~~~~~~~~~~~~~~~~~~~~~~~~~**

Rap1A cDNA **~~~~~~~~~~~~~~~~~~~~~~~~~~~~~~~~~~~~~~~~~~~~~~~~~~~~~~~~~~~~~~~~~~~~~~~~~~~~~~~~~~~~~~~~~~~~~~~~~~~~~~~~~~~~~~~~~~~~~~~~**

250 260 270 280 290 300 310 320 330 340 350 360

....|....|....|....|....|....|....|....|....|....|....|....|....|....|....|....|....|....|....|....|....|....|....|....|

Rap1A-retro1 cDNA **~~~~~~~~~~~~~~~~~~~~~~~~~~~~~~~~~~~~~~~~~~~~~~~~~~~~~~~~~~~~~~~~~~~~~~~~~~~~~~~~~~~~~~~~~~~~~~~~~~~~~~~~~~~~~~~~~~~~~~~~**

Rap1A-retro1 NCBI **AAAAATTTACCCAGAAAACAAAACTGAAAAACTTAGATTTTATCAATGCACCCTTTAAGTATTTTTTTCTTTTAGTTATTTTTTTTTCTTAAATTTTGAAAGCAGCTTAACGTGAATGAA**

Gtf2h1a **AAAAATTTACCCAGAAAACAAAACTGAAAAACTTAGATTTTATCAATGCACCCTTTAAGTATTTTTTTCTTTTAGTTATTTTTTTTTCTTAAATTTTGAAAGCAGCTTAACGTGAATGAA**

Rap1A-retro2 cDNA **~~~~~~~~~~~~~~~~~~~~~~~~~~~~~~~~~~~~~~~~~~~~~~~~~~~~~~~~~~~~~~~~~~~~~~~~~~~~~~~~~~~~~~~~~~~~~~~~~~~~~~~~~~~~~~~~~~~~~~~~**

Rap1A-retro2 csm **~~~~~~~~~~~~~~~~~~~~~~~~~~~~~~~~~~~~~~~~~~~~~~~~~~~~~~~~~~~~~~~~~~~~~~~~~~~~~~~~~~~~~~~~~~~~~~~~~~~~~~~~~~~~~~~~~~~~~~~~**

Rap1A-retro2 cel **AAAAATTTACCCAGAAAACAAAACTGAAAAACTTAGATTTTATCAATGCACCCTTTAAGTATTTTTTTCTTTTAGTTATTTTTTTT~CTTAAATTTTGAAAGCAGCTTAACGTGAATGAA**

GtF2h1b **~~~~~~~~~~~~~~~~~~~~~~~~~~~~~~~~~~~~~~~~~~~~~~~GCACCCTTTAAGTATTTTTTTCTTTTAGTTATTTTTTTT~CTTAAATTTTGAAAGCAGCTTAACGTGAATGAA**

Rap1A cDNA **~~~~~~~~~~~~~~~~~~~~~~~~~~~~~~~~~~~~~~~~~~~~~~~~~~~~~~~~~~~~~~~~~~~~~~~~~~~~~~~~~~~~~~~~~~~~~~~~~~~~~~~~~~~~~~~~~~~~~~~~**

370 380 390 400 410 420 430 440 450 460 470 480

....|....|....|....|....|....|....|....|....|....|....|....|....|....|....|....|....|....|....|....|....|....|....|....|

Rap1A-retro1 cDNA **~~~~~~~~~~~~~~~~~~~~~~~~~~~~~~~~~~~~~~~~~~~~~~~~~~~~~~~~~~~~~~~~~~~~~~~~~~~~~~~~~~~~~~~~~~~~~~~~~~~~~~~~~~~~~~~~~~~~~~~~**

Rap1A-retro1 NCBI **AAGCAGTTCCTGAGTAACTGCAGTAAACAGTCTTTAAATACATATACATGTACACATACATATATATTTGCAACTTAGCTCATTTTGGTTCTGCCTTGGTTTCCCTCCCCAGAGTTAGGT**

Gtf2h1a **AAGCAGTTCCTGAGTAACTGCAGTAAACAGTCTTTAAATACATATACATGTACACATACATATATATTTGCAACTTAGCTCATTTTGGTTCTGCCTTGGTTTCCCTCCCCAGAGTTAGGT**

Rap1A-retro2 cDNA **~~~~~~~~~~~~~~~~~~~~~~~~~~~~~~~~~~~~~~~~~~~~~~~~~~~~~~~~~~~~~~~~~~~~~~~~~~~~~~~~~~~~~~~~~~~~~~~~~~~~~~~~~~~~~~~~~~~~~~~~**

Rap1A-retro2 csm **~~~~~~~~~~~~~~~~~~~~~~~~~~~~~~~~~~~~~~~~~~~~~~~~~~~~~~~~~~~~~~~~~~~~~~~~~~~~~~~~~~~~~~~~~~~~~~~~~~~~~~~~~~~~~~~~~~~~~~~~**

Rap1A-retro2 cel **AAGCAGTTCCTGAGTAACTGCAGTAAACAGTCTTTAAATACATATACATGTACACATACATATATATTTGCAACTTAGCTCATTTTGGTTCTGCCTTGGTTTCCCTCCCCAGAGTTAGGT**

GtF2h1b **AAGCAGTTCCTGAGTAACTGCAGTAAACAGTCTTTAAATACATATACATGTACACATACATATATATTTGCAACTTAGCTCATTTTGGTTCTGCCTTGGTTTCCCTCCCCAGAGTTAGGT**

Rap1A cDNA **~~~~~~~~~~~~~~~~~~~~~~~~~~~~~~~~~~~~~~~~~~~~~~~~~~~~~~~~~~~~~~~~~~~~~~~~~~~~~~~~~~~~~~~~~~~~~~~~~~~~~~~~~~~~~~~~~~~~~~~~**

490 500 510 520 530 540 550 560 570 580 590 600

....|....|....|....|....|....|....|....|....|....|....|....|....|....|....|....|....|....|....|....|....|....|....|....|

Rap1A-retro1 cDNA **~~~~~~~~~~~~~~~~~~~~~~~~~~~~~~~~~~~~~~~~~~~~~~~~~~~~~~~~~~~~~~~~~~~~~~~~~~~~~~~~~~~~~~~~~~~~~~~~~~~~~~~~~~~~~~~~~~~~~~~~**

Rap1A-retro1 NCBI **GAATCCCGTCCCTTCCTTCT~~GTTTCAGTTTTCATGTG~CAAGTACAACAGCATCAGCTCTGGGAGTCGGCGGGCGTGCTGCAGATGTTTCCCCCGGTGTGAGCATCTGCTTGTCTTCA**

Gtf2h1a **GAATCCCGTCCCTTCCTTCT~~GTTTCAGTTTTCATGTG~CAAGTACAACAGCATCAGCTCTGGGAGTCGGCGGGCGTGCTGCAGATGTTTCCCCCGGTGTGAGCATCTGCTTGTCTTCA**

Rap1A-retro2 cDNA **~~~~~~~~~~~~~~~~~~~~~~~~~~~~~~~~~~~~~~~~~~~~~~~~~~~~~~~~~~~~~~~~~~~~~~~~~~~~~~~~~~~~~~~~~~~~~~~~~~~~~~~~~~~~~~~~~~~~~~~~**

Rap1A-retro2 csm **~~~~~~~~~~~~~~~~~~~~~~~~~~~~~~~~~~~~~~~~~~~~~~~~~~~~~~~~~~~~~~~~~~~~~~~~~~~~~~~~~~~~~~~~~~~~~~~~~~~~~~~~~~~~~~~~~~~~~~~~**

Rap1A-retro2 cel **GAATCCCGTCCCTTCCTTCTTTGTTTCAGTTCTCATGTG~CAAGTACAACAGCATCAGCTCTGGGAGTCGGCGGGCGCGCTGCAGATGTTTCCCC~GGTGTGAGCATCTGCTTGTCTTCA**

GtF2h1b **GAATCCCGTCCCTTCCTTCTTTGTTTCAGTTCTCATGTGCCAAGTACAACAGCATCAGCTCTGGGAGTCGGCGGGCGCGCTGCAGATGTTTCCCC~GGTGTGAGCATCTGCTTGTCTTCA**

Rap1A cDNA **~~~~~~~~~~~~~~~~~~~~~~~~~~~~~~~~~~~~~~~~~~~~~~~~~~~~~~~~~~~~~~~~~~~~~~~~~~~~~~~~~~~~~~~~~~~~~~~~~~~~~~~~~~~~~~~~~~~~~~~~**

610 620 630 640 650 660 670 680 690 700 710 720

....|....|....|....|....|....|....|....|....|....|....|....|....|....|....|....|....|....|....|....|....|....|....|....|

Rap1A-retro1 cDNA **~~~~~~~~~~~~~~~~~~~~~~~~~~~~~~~~~~~~~~~~~~~~~~~~~~~~~~~~~~~~~~~~~~~~~~~~~~~~~~~~~~~~~~~~~~~~~~~~~~~~~~~~~~~~~~~~~~~~~~~~**

Rap1A-retro1 NCBI **GCCAGGTTTCCAGAGTGACTGCAGGTAAGTTTTTTTTCAACCCACAAAACCTGTGAGCACCATAAACGCTTCACGTCTTCTTCATCAGGCGCCGGGATTGCCACGCGTGGAGCTTGTTGT**

Gtf2h1a **GCCAGGTTTCCAGAGTGACTGCAGGTAAGTTTTTTTTCAACCCACAAAACCTGTGAGCACCATAAACGCTTCACGTCTTCTTCATCAGGCGCCGGGATTGCCACGCGTGGAGCTTGTTGT**

Rap1A-retro2 cDNA **~~~~~~~~~~~~~~~~~~~~~~~~~~~~~~~~~~~~~~~~~~~~~~~~~~~~~~~~~~~~~~~~~~~~~~~~~~~~~~~~~~~~~~~~~~~~~~~~~~~~~~~~~~~~~~~~~~~~~~~~**

Rap1A-retro2 csm **~~~~~~~~~~~~~~~~~~~~~~~~~~~~~~~~~~~~~~~~~~~~~~~~~~~~~~~~~~~~~~~~~~~~~~~~~~~~~~~~~~~~~~~~~~~~~~~~~~~~~~~~~~~~~~~~~~~~~~~~**

Rap1A-retro2 cel **GCCAGGTTTCCAGAGTGACTGCAGGTCAGTTTTTTTTCAACCCACAAAACCTGTGAGCACCATAAACGCTTCACGTCTTCTTCATCAGGCGCCGGGATTGCCATGTGTGGAGCTTGTTGT**

GtF2h1b **GCCAGGTTTCCAGAGTGACTGCAGGTCAGTTTTTTTTCAACCCACAAAACCTGTGAGCACCATAAACGCTTCACGTCTTCTTCATCAGGCGCCGGGATTGCCATGTGTGGAGCTTGTTGT**

Rap1A cDNA **~~~~~~~~~~~~~~~~~~~~~~~~~~~~~~~~~~~~~~~~~~~~~~~~~~~~~~~~~~~~~~~~~~~~~~~~~~~~~~~~~~~~~~~~~~~~~~~~~~~~~~~~~~~~~~~~~~~~~~~~**

730 740 750 760 770 780 790 800 810 820 830 840

....|....|....|....|....|....|....|....|....|....|....|....|....|....|....|....|....|....|....|....|....|....|....|....|

Rap1A-retro1 cDNA **~~~~~~~~~~~~~~~~~~~~~~~~~~~~~~~~~~~~~~~~~~~~~~~~~~~~~~~~~~~~~~~~~~~~~~~~~~~~~~~~~~~~~~~~~~~~~~~~~~~~~~~~~~~~~~~~~~~~~~~~**

Rap1A-retro1 NCBI **AGGCTGTCTGCAGCATCTCTTCTATGTGACTTACCAGCTGCAAGAGAC~~~~~~~~~~~~~AGTGAGTAGTCAGGGAAG~~~~CCACTGCTTCACAGCTCAGCCTGGCACACACTTTCCA**

Gtf2h1a **AGGCTGTCTGCAGCATCTCTTCTATGTGACTTACCAG~~~~~~~~~~~~~~~~~~~~~~~~~~~~~~~~~~~~~~~~~~~~~~~~~~~~~~~~~~~~~~~~~~~~~~~~~~~~~~~~~~~**

Rap1A-retro2 cDNA **~~~~~~~~~~~~~~~~~~~~~~~~~~~~~~~~~~~~~~~~~~~~~~~~~~~~~~~~~~~~~~~~~~~~~~~~~~~~~~~~~~~~~~~~~~~~~~~~~~~~~~~~~~~~~~~~~~~~~~~~**

Rap1A-retro2 csm **~~~~~~~~~~~~~~~~~~~~~~~~~~~~~~~~~~~~~~~~~~~~~~~~~~~~~~~~~~~~~~~~~~~~~~~~~~~~~~~~~~~~~~~~~~~~~~~~~~~~~~~~~~~~~~~~~~~~~~~~**

Rap1A-retro2 cel **AGGCTGTCTGCAGCATCTCTTCTATGTGACTTACCAGCTGCAAGAGACAGGGAGATACTGCAGTGAGTAGTCAGGGAAGCAAGCCACTGCTTCACAGCTCAGCCTGGCACACACTTTCCA**

GtF2h1b **AGGCTGTCTGCAGCATCTCTTCTATGTGACTTACCAG~~~~~~~~~~~~~~~~~~~~~~~~~~~~~~~~~~~~~~~~~~~~~~~~~~~~~~~~~~~~~~~~~~~~~~~~~~~~~~~~~~~**

Rap1A cDNA **~~~~~~~~~~~~~~~~~~~~~~~~~~~~~~~~~~~~~~~~~~~~~~~~~~~~~~~~~~~~~~~~~~~~~~~~~~~~~~~~~~~~~~~~~~~~~~~~~~~~~~~~~~~~~~~~~~~~~~~~**

850 860 870 880 890 900 910 920 930 940 950 960

....|....|....|....|....|....|....|....|....|....|....|....|....|....|....|....|....|....|....|....|....|....|....|....|

Rap1A-retro1 cDNA **~~~~~~~~~~~~~~~~~~~~~~~~~~~~~~~~~~~~~~~~~~~~~~~~~~~~~~~~~~~~~~~~~~~~~~~~~~~~~~~~~~~~~~~~~~~~~~~~~~~~~~~~~~~~~~~~~~~~~~~~**

Rap1A-retro1 NCBI **GCACATCACTCAGTCAGCAAGGCGTGTGTGTATAAACCCTCAAGCAAAGGTGTGACCACACACTGTGCATGTGCAAGATAGGTGGTAGGGGCAGTGCAGGATCTGGACAGTCACCCGCTC**

Gtf2h1a **~~~~~~~~~~~~~~~~~~~~~~~~~~~~~~~~~~~~~~~~~~~~~~~~~~~~~~~~~~~~~~~~~~~~~~~~~~~~~~~~~~~~~~~~~~~~~~~~~~~~~~~~~~~~~~~~~~~~~~~~**

Rap1A-retro2 cDNA **~~~~~~~~~~~~~~~~~~~~~~~~~~~~~~~~~~~~~~~~~~~~~~~~~~~~~~~~~~~~~~~~~~~~~~~~~~~~~~~~~~~~~~~~~~~~~~~~~~~~~~~~~~~~~~~~~~~~~~~~**

Rap1A-retro2 csm **~~~~~~~~~~~~~~~~~~~~~~~~~~~~~~~~~~~~~~~~~~~~~~~~~~~~~~~~~~~~~~~~~~~~~~~~~~~~~~~~~~~~~~~~~~~~~~~~~~~~~~~~~~~~~~~~~~~~~~~~**

Rap1A-retro2 cel **GCACATCTCTCAGTCAGCAAGGCGTGTGTGTATAAACCCTCAAGCAAAGGTGTGACCACACACTGTGCATGCGCAAGATAGGTGGTAGGGGCAGTGCAGGATTGGGACAGTCACCCGCTC**

GtF2h1b **~~~~~~~~~~~~~~~~~~~~~~~~~~~~~~~~~~~~~~~~~~~~~~~~~~~~~~~~~~~~~~~~~~~~~~~~~~~~~~~~~~~~~~~~~~~~~~~~~~~~~~~~~~~~~~~~~~~~~~~~**

Rap1A cDNA **~~~~~~~~~~~~~~~~~~~~~~~~~~~~~~~~~~~~~~~~~~~~~~~~~~~~~~~~~~~~~~~~~~~~~~~~~~~~~~~~~~~~~~~~~~~~~~~~~~~~~~~~~~~~~~~~~~~~~~~~**

970 980 990 1000 1010 1020 1030 1040 1050 1060 1070 1080

....|....|....|....|....|....|....|....|....|....|....|....|....|....|....|....|....|....|....|....|....|....|....|....|

Rap1A-retro1 cDNA **~~~~~~~~~~~~~~~~~~~~~~~~~~~~~~~~~~~~~~~~~~~~~~~~~~~~~~~~~~~~~~~~~~~~~~~~~~~~~~~~~~~~~~~~~~~~~~~~~~~~~~~~~~~~~~~~~~~~~~~~**

Rap1A-retro1 NCBI **TATCTGGGTTATGATACTAGGCCTGGCCATTAACTAGCACCAGCACCAGAACTGCTTTTGTGCAATGAGAGCCAAACCAGGTTGATTTCTTGATTTATACACTGGTCAGCTATTGAAAGT**

Gtf2h1a **~~~~~~~~~~~~~~~~~~~~~~~~~~~~~~~~~~~~~~~~~~~~~~~~~~~~~~~~~~~~~~~~~~~~~~~~~~~~~~~~~~~~~~~~~~~~~~~~~~~~~~~~~~~~~~~~~~~~~~~~**

Rap1A-retro2 cDNA **~~~~~~~~~~~~~~~~~~~~~~~~~~~~~~~~~~~~~~~~~~~~~~~~~~~~~~~~~~~~~~~~~~~~~~~~~~~~~~~~~~~~~~~~~~~~~~~~~~~~~~~~~~~~~~~~~~~~~~~~**

Rap1A-retro2 csm **~~~~~~~~~~~~~~~~~~~~~~~~~~~~~~~~~~~~~~~~~~~~~~~~~~~~~~~~~~~~~~~~~~~~~~~~~~~~~~~~~~~~~~~~~~~~~~~~~~~~~~~~~~~~~~~~~~~~~~~~**

Rap1A-retro2 cel **TATCTGGGTTATGATACTATGCCTGGCCATTAACCAGCACCAGCACCAGAATTGCTTTTGTGCAATGAGAGCCAAACCAGGTTGATTTCTTGATTTATACACTGGTCAGCTATTGAAAGT**

GtF2h1b **~~~~~~~~~~~~~~~~~~~~~~~~~~~~~~~~~~~~~~~~~~~~~~~~~~~~~~~~~~~~~~~~~~~~~~~~~~~~~~~~~~~~~~~~~~~~~~~~~~~~~~~~~~~~~~~~~~~~~~~~**

Rap1A cDNA **~~~~~~~~~~~~~~~~~~~~~~~~~~~~~~~~~~~~~~~~~~~~~~~~~~~~~~~~~~~~~~~~~~~~~~~~~~~~~~~~~~~~~~~~~~~~~~~~~~~~~~~~~~~~~~~~~~~~~~~~**

1090 1100 1110 1120 1130 1140 1150 1160 1170 1180 1190 1200

....|....|....|....|....|....|....|....|....|....|....|....|....|....|....|....|....|....|....|....|....|....|....|....|

Rap1A-retro1 cDNA **~~~~~~~~~~~~~~~~~~~~~~~~~~~~~~~~~~~~~~~~~~~~~~~~~~~~~~~~~~~~~~~~~~~~~~~~~~~~~~~~~~~~~~~~~~~~~~~~~~~~~~~~~~~~~~~~~~~~~~~~**

Rap1A-retro1 NCBI **CAGGACAGCAACAGCAACACTTTTTCTCTGCGAAGACCCTCAGTCTGGCTTTGAACTTATAGCAGCCTCCCTGCCCCTGCCTGAGTGCCAGGACCATTGGCACTCACTGTCAAACCCGAC**

Gtf2h1a **~~~~~~~~~~~~~~~~~~~~~~~~~~~~~~~~~~~~~~~~~~~~~~~~~~~~~~~~~~~~~~~~~~~~~~~~~~~~~~~~~~~~~~~~~~~~~~~~~~~~~~~~~~~~~~~~~~~~~~~~**

Rap1A-retro2 cDNA **~~~~~~~~~~~~~~~~~~~~~~~~~~~~~~~~~~~~~~~~~~~~~~~~~~~~~~~~~~~~~~~~~~~~~~~~~~~~~~~~~~~~~~~~~~~~~~~~~~~~~~~~~~~~~~~~~~~~~~~~**

Rap1A-retro2 csm **~~~~~~~~~~~~~~~~~~~~~~~~~~~~~~~~~~~~~~~~~~~~~~~~~~~~~~~~~~~~~~~~~~~~~~~~~~~~~~~~~~~~~~~~~~~~~~~~~~~~~~~~~~~~~~~~~~~~~~~~**

Rap1A-retro2 cel **CAGGACAGCAACAGCAACACTTTTTCTCTGCGAAGACCCTCAGTCTGGCTTTGAACTTATAGCAGCCTCCCTGCCCCTGCCTGAGTGCCAGGACCATTGGTACTCACTGTCAAACCCGAC**

GtF2h1b **~~~~~~~~~~~~~~~~~~~~~~~~~~~~~~~~~~~~~~~~~~~~~~~~~~~~~~~~~~~~~~~~~~~~~~~~~~~~~~~~~~~~~~~~~~~~~~~~~~~~~~~~~~~~~~~~~~~~~~~~**

Rap1A cDNA **~~~~~~~~~~~~~~~~~~~~~~~~~~~~~~~~~~~~~~~~~~~~~~~~~~~~~~~~~~~~~~~~~~~~~~~~~~~~~~~~~~~~~~~~~~~~~~~~~~~~~~~~~~~~~~~~~~~~~~~~**

1210 1220 1230 1240 1250 1260 1270 1280 1290 1300 1310 1320

....|....|....|....|....|....|....|....|....|....|....|....|....|....|....|....|....|....|....|....|....|....|....|....|

Rap1A-retro1 cDNA **~~~~~~~~~~~~~~~~~~~~~~~~~~~~~~~~~~~~~~~~~~~~~~~~~~~~~~~~~~~~~~~~~~~~~~~~~~~~~~~~~~~~~~~~~~~~~~~~~~~~~~~~~~~~~~~~~~~~~~~~**

Rap1A-retro1 NCBI **TCAACGCTGAACTTT~~~~~~~~~GGAGAGTTCTGCCTTCGGGGGTCTGACAAAGTCAAGTAAGAGGAGCACACTAACCCTTGAGGTCAGAAAGCCTTGGAGTATGTTTACGTGGTTACA**

Gtf2h1a **~~~~~~~~~~~~~~~~~~~~~~~~~~~~~~~~~~~~~~~~~~~~~~~~~~~~~~~~~~~~~~~~~~~~~~~~~~~~~~~~~~~~~~~~~~~~~~~~~~~~~~~~~~~~~~~~~~~~~~~~**

Rap1A-retro2 cDNA **~~~~~~~~~~~~~~~~~~~~~~~~~~~~~~~~~~~~~~~~~~~~~~~~~~~~~~~~~~~~~~~~~~~~~~~~~~~~~~~~~~~~~~~~~~~~~~~~~~~~~~~~~~~~~~~~~~~~~~~~**

Rap1A-retro2 csm **~~~~~~~~~~~~~~~~~~~~~~~~~~~~~~~~~~~~~~~~~~~~~~~~~~~~~~~~~~~~~~~~~~~~~~~~~~~~~~~~~~~~~~~~~~~~~~~~~~~~~~~~~~~~~~~~~~~~~~~~**

Rap1A-retro2 cel **TCAACGCTGAACTTTTACTACTTTGGAGAGTTCTGCCTTCGGGGGTCTGACAAAGTCAAGTAAGAGGAGCACACTAACCCTTGAGGTCAGAAAGCCTTGGAGTATGTTTACGTGGTTACA**

GtF2h1b **~~~~~~~~~~~~~~~~~~~~~~~~~~~~~~~~~~~~~~~~~~~~~~~~~~~~~~~~~~~~~~~~~~~~~~~~~~~~~~~~~~~~~~~~~~~~~~~~~~~~~~~~~~~~~~~~~~~~~~~~**

Rap1A cDNA **~~~~~~~~~~~~~~~~~~~~~~~~~~~~~~~~~~~~~~~~~~~~~~~~~~~~~~~~~~~~~~~~~~~~~~~~~~~~~~~~~~~~~~~~~~~~~~~~~~~~~~~~~~~~~~~~~~~~~~~~**

1330 1340 1350 1360 1370 1380 1390 1400 1410 1420 1430 1440

....|....|....|....|....|....|....|....|....|....|....|....|....|....|....|....|....|....|....|....|....|....|....|....|

Rap1A-retro1 cDNA **~~~~~~~~~~~~~~~~~~~~~~~~~~~~~~~~~~~~~~~~~~~~~~~~~~~~~~~~~~~~~~~~~~~~~~~~~~~~~~~~~~~~~~~~~~~~~~~~~~~~~~~~~~~~~~~~~~~~~~~~**

Rap1A-retro1 NCBI **GTAAAGTCTTTCAAGTTTATCATTCTGGGAGGGAGGGGGTGGACTTGGGGTGCTCTAAGTCACTGATTTTGCTCTAGGAGAGAGATTTAAGTATGCCCCTACCCCAGCTGCATATGCAGG**

Gtf2h1a **~~~~~~~~~~~~~~~~~~~~~~~~~~~~~~~~~~~~~~~~~~~~~~~~~~~~~~~~~~~~~~~~~~~~~~~~~~~~~~~~~~~~~~~~~~~~~~~~~~~~~~~~~~~~~~~~~~~~~~~~**

Rap1A-retro2 cDNA **~~~~~~~~~~~~~~~~~~~~~~~~~~~~~~~~~~~~~~~~~~~~~~~~~~~~~~~~~~~~~~~~~~~~~~~~~~~~~~~~~~~~~~~~~~~~~~~~~~~~~~~~~~~~~~~~~~~~~~~~**

Rap1A-retro2 csm **~~~~~~~~~~~~~~~~~~~~~~~~~~~~~~~~~~~~~~~~~~~~~~~~~~~~~~~~~~~~~~~~~~~~~~~~~~~~~~~~~~~~~~~~~~~~~~~~~~~~~~~~~~~~~~~~~~~~~~~~**

Rap1A-retro2 cel **GTAAAGTCTTTCAAGTTTATCATTCTGGGAGGAAGGGGGTGGACTTGGGGTGCTCTAAGTCAGTGATTTTGCTCTAGGAGAGAGATTTAAGTATGCCCTTACCCCAGCTGCACATGCAGG**

GtF2h1b **~~~~~~~~~~~~~~~~~~~~~~~~~~~~~~~~~~~~~~~~~~~~~~~~~~~~~~~~~~~~~~~~~~~~~~~~~~~~~~~~~~~~~~~~~~~~~~~~~~~~~~~~~~~~~~~~~~~~~~~~**

Rap1A cDNA **~~~~~~~~~~~~~~~~~~~~~~~~~~~~~~~~~~~~~~~~~~~~~~~~~~~~~~~~~~~~~~~~~~~~~~~~~~~~~~~~~~~~~~~~~~~~~~~~~~~~~~~~~~~~~~~~~~~~~~~~**

1450 1460 1470 1480 1490 1500 1510 1520 1530 1540 1550 1560

....|....|....|....|....|....|....|....|....|....|....|....|....|....|....|....|....|....|....|....|....|....|....|....|

Rap1A-retro1 cDNA **~~~~~~~~~~~~~~~~~~~~~~~~~~~~~~~~~~~~~~~~~~~~~~~~~~~~~~~~~~~~~~~~~~~~~~~~~~~~~~~~~~~~~~~~~~~~~~~~~~~~~~~~~~~~~~~~~~~~~~~~**

Rap1A-retro1 NCBI **ACAGAAGCAGAGCACTTC~~~~~~~TGTGTAAAGTGCACATTCAATCTTCAGCAATCCCCAGTTCTAATCCCCTTTTCCCGGAGGAGGGTGTGGCACTAGCTTTGTCTGGAAAATGTAGC**

Gtf2h1a **~~~~~~~~~~~~~~~~~~~~~~~~~~~~~~~~~~~~~~~~~~~~~~~~~~~~~~~~~~~~~~~~~~~~~~~~~~~~~~~~~~~~~~~~~~~~~~~~~~~~~~~~~~~~~~~~~~~~~~~~**

Rap1A-retro2 cDNA **~~~~~~~~~~~~~~~~~~~~~~~~~~~~~~~~~~~~~~~~~~~~~~~~~~~~~~~~~~~~~~~~~~~~~~~~~~~~~~~~~~~~~~~~~~~~~~~~~~~~~~~~~~~~~~~~~~~~~~~~**

Rap1A-retro2 csm **~~~~~~~~~~~~~~~~~~~~~~~~~~~~~~~~~~~~~~~~~~~~~~~~~~~~~~~~~~~~~~~~~~~~~~~~~~~~~~~~~~~~~~~~~~~~~~~~~~~~~~~~~~~~~~~~~~~~~~~~**

Rap1A-retro2 cel **ACAGAAGCAGAGCACCTGCCTAGAATGTGTAAAGTGCACATTCCATCTTCAGCAATCCCCAGGTCTAATCCCCTTTTCCCGGGGGAGGGTGTGGCACTGGCTTTGTCTGGAAAATGTAGC**

GtF2h1b **~~~~~~~~~~~~~~~~~~~~~~~~~~~~~~~~~~~~~~~~~~~~~~~~~~~~~~~~~~~~~~~~~~~~~~~~~~~~~~~~~~~~~~~~~~~~~~~~~~~~~~~~~~~~~~~~~~~~~~~~**

Rap1A cDNA **~~~~~~~~~~~~~~~~~~~~~~~~~~~~~~~~~~~~~~~~~~~~~~~~~~~~~~~~~~~~~~~~~~~~~~~~~~~~~~~~~~~~~~~~~~~~~~~~~~~~~~~~~~~~~~~~~~~~~~~~**

1570 1580 1590 1600 1610 1620 1630 1640 1650 1660 1670 1680

....|....|....|....|....|....|....|....|....|....|....|....|....|....|....|....|....|....|....|....|....|....|....|....|

Rap1A-retro1 cDNA **~~~~CCTTTCTGCTTGACCGGTAGCGATGGCTTTACAGTACCAGTGAGATGTATTTCATATGCTTCCATATGGCTACACATAGCCTGTGAATCCTCAACACAGTGCTGTAAACACAATCA**

Rap1A-retro1 NCBI **AATTCCTTTCTGCTTGACCGGTAGCGATGGCTTTACAGTACCAGTGAGATGTATTTCATATGCTTCCATATGGCTACACATAGCCTGTGAATCCTCAACACAGTGCTGTAAACACAATCA**

Gtf2h1a **~~~~~~~~~~~~~~~~~~~~~~~~~~~~~~~~~~~~~~~~~~~~~~~~~~~~~~~~~~~~~~~~~~~~~~~~~~~~~~~~~~~~~~~~~~~~~~~~~~~~~~~~~~~~~~~~~~~~~~~~**

Rap1A-retro2 cDNA **~~~~~~~~~~~~~~~~~~~~~~~~~~~~~~~~~~~~~~~~~~~~~~~~~~~~~~~~~~~~~~~~~~~~~~~~~~~~~~~~~~~~~~~~~~~~~~~~~~~~~~~~~~~~~~~~~~~~~~~~**

Rap1A-retro2 csm **~~~~~~~~~~~~~~~~~~~~~~~~~~~~~~~~~~~~~~~~~~~~~~~~~~~~~~~~~~~~~~~~~~~~~~~~~~~~~~~~~~~~~~~~~~~~~~~~~~~~~~~~~~~~~~~~~~~~~~~~**

Rap1A-retro2 cel **AATTCCTTTCTGCTTGACTGGTAGCGGTGGCTTTACAGTACCAGTGAGATGTAGTTCATATGCTTCGATATGGCTACAAAGATCCTGTGAATCCTCAACACAGTGCTGTAAACACAATCA**

GtF2h1b **~~~~~~~~~~~~~~~~~~~~~~~~~~~~~~~~~~~~~~~~~~~~~~~~~~~~~~~~~~~~~~~~~~~~~~~~~~~~~~~~~~~~~~~~~~~~~~~~~~~~~~~~~~~~~~~~~~~~~~~~**

Rap1A cDNA **~~~~~~~~~~~~~~~~~~~~~~~~~~~~~~~~~~~~~~~~~~~~~~~~~~~~~~~~~~~~~~~~~~~~~~~~~~~~~~~~~~~~~~~~~~~~~~~~~~~~~~~~~~~~~~~~~~~~~~~~**

1690 1700 1710 1720 1730 1740 1750 1760 1770 1780 1790 1800

....|....|....|....|....|....|....|....|....|....|....|....|....|....|....|....|....|....|....|....|....|....|....|....|

Rap1A-retro1 cDNA **ACCGAGGCGGCACAAAAGGCAGAGAACCTGCTTAGTGTTCTTGGCCTGCTGTAAGAGACACTCAGTGCTCACTGGGGAAAATCATAAGTCATCAAGATTTTGCTAAACCCCATTTCTGAT**

Rap1A-retro1 NCBI **ACCGAGGCGGCACAAAAGGCAGAGAACCTGCTTAGTGTTCTTGGCCTGCTGTAAGAGACACTCAGTGCTCACTGGGGAAAATCATAAGTCATCAAGATTTTGCTAAACCCCATTTCTGAT**

Gtf2h1a **~~~~~~~~~~~~~~~~~~~~~~~~~~~~~~~~~~~~~~~~~~~~~~~~~~~~~~~~~~~~~~~~~~~~~~~~~~~~~~~~~~~~~~~~~~~~~~~~~~~~~~~~~~~~~~~~~~~~~~~~**

Rap1A-retro2 cDNA **~~~~~~~~~~~~~~~~~~~~~~~~~~~~~~~~~~~~~~~~~~~~~~~~~~~~~~~~~~~~~~~~~~~~~~~~~~~~~~~~~~~~~~~~~~~~~~~~~~~~~~~~~~~~~~~~~~~~~~~~**

Rap1A-retro2 csm **~~~~~~~~~~~~~~~~~~~~~~~~~~~~~~~~~~~~~~~~~TGGCCTGCTGTAAGAGACACTCAGGGCTCACTGGGGAAAATCATAAATCATCAAGATTTTGCTAAACCCCATTTCTGAT**

Rap1A-retro2 cel **ACCGAGGCGGCACAAAAGGCAGAGAACCTGCTTAGTGTTCTTGGCCTGCTGTAAGAGACACTCAGGGCTCACTGGGGAAAATCATAAATCATCAAGATTTTGCTAAACCCCATTTCTGAT**

GtF2h1b **~~~~~~~~~~~~~~~~~~~~~~~~~~~~~~~~~~~~~~~~~~~~~~~~~~~~~~~~~~~~~~~~~~~~~~~~~~~~~~~~~~~~~~~~~~~~~~~~~~~~~~~~~~~~~~~~~~~~~~~~**

Rap1A cDNA **~~~~~~~~~~~~~~~~~~~~~~~~~~~~~~~~~~~~~~~~~~~~~~~~~~~~~~~~~~~~~~~~~~~~~~~~~~~~~~~~~~~~~~~~~~~~~~~~~~~~~~~~~~~~~~~~~~~~~~~~**

1810 1820 1830 1840 1850 1860 1870 1880 1890 1900 1910 1920

....|....|....|....|....|....|....|....|....|....|....|....|....|....|....|....|....|....|....|....|....|....|....|....|

Rap1A-retro1 cDNA **CATCCTAGTCATAGCTAGATGTTATGTTTGCATATACTATTTCTCAGAAAGTCAGTTTCTCTCGAAGAAACAAACCTCTTCAACATTTGTTCTAGCTCTGCTTGGAAACAGTACTAAAGA**

Rap1A-retro1 NCBI **CATCCTAGTCATAGCTAGATGTTATGTTTGCATATACTATTTCTCAGAAAGTCAGTTTCTCTCGAAGAAACAAACCTCTTCAACATTTGTTCTAGCTCTGCTTGGAAACAGTACTAAAGA**

Gtf2h1a **~~~~~~~~~~~~~~~~~~~~~~~~~~~~~~~~~~~~~~~~~~~~~~~~~~~~~~~~~~~~~~~~~~~~~~~~~~~~~~~~~~~~~~~~~~~~~~~~~~~~~~~~~~~~~~~~~~~~~~~~**

Rap1A-retro2 cDNA **~~~~~~~~~~~~~~~~~~~~~~~~~~~~~~~~~~~~~~~~~~~~~~~~~~~~~~~~~~~~~~~~~~~~~~~~~~~~~~~~~~~~~~~~~~~~~~~~~~~~~~~~~~~~~~~~~~~~~~~~**

Rap1A-retro2 csm **CATCCTAGTCATAGCTAGACGTTATGTTTGCATATACTATTTCTCAGAAAGTCAGTTTCTCTCGAAGAAACAAACCTCTTCAACATTTGTTCTAGCTCTGCTTGGAAACAATACTAAAGA**

Rap1A-retro2 cel **CATCCTAGTCATAGCTAGACGTTATGTTTGCATATACTATTTCTCAGAAAGTCAGTTTCTCTCGAAGAAACAAACCTCTTCAACATTTGTTCTAGCTCTGCTTGGAAACAATACTAAAGA**

GtF2h1b **~~~~~~~~~~~~~~~~~~~~~~~~~~~~~~~~~~~~~~~~~~~~~~~~~~~~~~~~~~~~~~~~~~~~~~~~~~~~~~~~~~~~~~~~~~~~~~~~~~~~~~~~~~~~~~~~~~~~~~~~**

Rap1A cDNA **~~~~~~~~~~~~~~~~~~~~~~~~~~~~~~~~~~~~~~~~~~~~~~~~~~~~~~~~~~~~~~~~~~~~~~~~~~~~~~~~~~~~~~~~~~~~~~~~~~~~~~~~~~~~~~~~~~~~~~~~**

1930 1940 1950 1960 1970 1980 1990 2000 2010 2020 2030 2040

....|....|....|....|....|....|....|....|....|....|....|....|....|....|....|....|....|....|....|....|....|....|....|....|

Rap1A-retro1 cDNA **CCTTGTGCAAACCAACCCTGCTCCACCACTGGCCTTATTGCTGTAGAGCCCCATCCAAGGACAGCCCTGCTTCCTATCATTAACATTTTACCTGCCTATCACTGCCAGGTTTGTAGTAAC**

Rap1A-retro1 NCBI **CCTTGTGCAAACCAACCCTGCTCCACCACTGGCCTTATTGCTGTAGAGCCCCATCCAAGGACAGCCCTGCTTCCTATCATTAACATTTTACCTGCCTATCACTGCCAGGTTTGTAGTAAC**

Gtf2h1a **~~~~~~~~~~~~~~~~~~~~~~~~~~~~~~~~~~~~~~~~~~~~~~~~~~~~~~~~~~~~~~~~~~~~~~~~~~~~~~~~~~~~~~~~~~~~~~~~~~~~~~~~~~~~~~~~~~~~~~~~**

Rap1A-retro2 cDNA **~~~~~~~~~~~~~~~~~~~~~~~~~~~~~~~~~~~~~~~~~~~~~~~~~~~~~~~~~~~~~~~~~~~~~~~~~~~~~~~~~~~~~~~~~~~~~~~~~~~~~~~~~~~~~~~~~~~~~~~~**

Rap1A-retro2 csm **CCTTGTGCAACCCAACCCTGCTCCACCACTGGCCTTATTGCTGTAGAGCACCATCCAAGGACAGCCCTGCTTCCGATCATTAACATTTTACCTGCCTATCACTGCCAGGTTTGTAGTAAC**

Rap1A-retro2 cel **CCTTGTGCAACCCAACCCTGCTCCACCACTGGCCTTATTGCTGTAGAGCACCATCCAAGGACAGCCCTGCTTCCGATCATTAACATTTTACCTGCCTATCACTGCCAGGTTTGTAGTAAC**

GtF2h1b **~~~~~~~~~~~~~~~~~~~~~~~~~~~~~~~~~~~~~~~~~~~~~~~~~~~~~~~~~~~~~~~~~~~~~~~~~~~~~~~~~~~~~~~~~~~~~~~~~~~~~~~~~~~~~~~~~~~~~~~~**

Rap1A cDNA **~~~~~~~~~~~~~~~~~~~~~~~~~~~~~~~~~~~~~~~~~~~~~~~~~~~~~~~~~~~~~~~~~~~~~~~~~~~~~~~~~~~~~~~~~~~~~~~~~~~~~~~~~~~~~~~~~~~~~~~~**

2050 2060 2070 2080 2090 2100 2110 2120 2130 2140 2150 2160

....|....|....|....|....|....|....|....|....|....|....|....|....|....|....|....|....|....|....|....|....|....|....|....|

Rap1A-retro1 cDNA **AGATTTTTTTAGGACTATATACTTAATAAATCTATCAAGAAAAACCAGAAAAAAA~~~~~~~GAAAAAGAAAAACCAGGGAGCCGCGGCCGAGAGGAGGGAGGAGGAGGAGGTGGAGGAG**

Rap1A-retro1 NCBI **AGATTTTTTTAGGACTATATACTTAATAAATCTATCAAGAAAAACCAGAAAAAAA~~~~~~~GAAAAAGAAAAACCAGGGAGCCGCGGCCGAGAGGAGGGAGGAGGAGGAGGTGGAGGAG**

Gtf2h1a **~~~~~~~~~~~~~~~~~~~~~~~~~~~~~~~~~~~~~~~~~~~~~~~~~~~~~~~~~~~~~~~~~~~~~~~~~~~~~~~~~~~~~~~~~~~~~~~~~~~~~~~~~~~~~~~~~~~~~~~~**

Rap1A-retro2 cDNA **~~~~~~~~~~~~~~~~~~~~~~~~~~~~~~~~~~~~~~~~~~~~~~~~~~~~~~~~~~~~~~~~~~~~~~~~~~~~~~~~~~~~~~~~~~~~~~~~~~~~~~~~~~~~~~~~~~~~~~~~**

Rap1A-retro2 csm **AGATTTTTTTAGGACTATATACTTAATAAATCTATCAAGAAAAAACAGAAAAAAAGAAAAAAGAAAAAGAAAAACCAGGGAGCCGCGGCCGAGAGGAGGGAGGAGGAGGAGGTGGAGGAG**

Rap1A-retro2 cel **AGATTTTTTTAGGACTATATACTTAATAAATCTATCAAGAAAAAACAGAAAAAAAGAAAAAAGAAAAAGAAAAACCAGGGAGCCGCGGCCGAGAGGAGGGAGGAGGAGGAGGTGGAGGAG**

GtF2h1b **~~~~~~~~~~~~~~~~~~~~~~~~~~~~~~~~~~~~~~~~~~~~~~~~~~~~~~~~~~~~~~~~~~~~~~~~~~~~~~~~~~~~~~~~~~~~~~~~~~~~~~~~~~~~~~~~~~~~~~~~**

Rap1A cDNA **~~~~~~~~~~~~~~~~~~~~~~~~~~~~~~~~~~~~~~~~~~~~~~~~~~~~~~~~~~~~~~~~~~~~~~~~~~~~~~~GAGCCGCGGCCGAGAGGAGGGAGGAGGAGGAGGTGGAGGAG**

2170 2180 2190 2200 2210 2220 2230 2240 2250 2260 2270 2280

....|....|....|....|....|....|....|....|....|....|....|....|....|....|....|....|....|....|....|....|....|....|....|....|

Rap1A-retro1 cDNA **GCGCCGGGCCGCGGGATTGTCAATATTTAAACAGAGCGCATCATGCGTGAGTACAAGCTAGTAGTCCTTGGTTCAGGAGGCGTGGGGAAGTCTGCTCTGACAGTTCAGTTTGTTCAGGGA**

Rap1A-retro1 NCBI **GCGCCGGGCCGCGGGATTGTCAATATTTAAACAGAGCGCATCATGCGTGAGTACAAGCTAGTAGTCCTTGGTTCAGGAGGCGTGGGGAAGTCTGCTCTGACAGTTCAGTTTGTTCAGGGA**

Gtf2h1a **~~~~~~~~~~~~~~~~~~~~~~~~~~~~~~~~~~~~~~~~~~~~~~~~~~~~~~~~~~~~~~~~~~~~~~~~~~~~~~~~~~~~~~~~~~~~~~~~~~~~~~~~~~~~~~~~~~~~~~~~**

Rap1A-retro2 cDNA **~~~~~~~~~~GCGGGATTGTCAATATTTAAACAGAGCGCATCATGCGTGAGTACAAGCTAGTAGTTATTGGTTCAGGAGGCGTGGGGAAGTCTGCTCTGACAGTTCAGTTTGTTCAGGGA**

Rap1A-retro2 csm **GCGCCGGGCCGCGGGATTGTCAATATTTAAACAGAGCGCATCATGCGTGAGTACAAGCTAGTAGTTATTGGTTCAGGAGGCGTGGGGAAGTCTGCTCTGACAGTTCAGTTTGTTCAGGGA**

Rap1A-retro2 cel **GCGCCGGGCCGCGGGATTGTCAATATTTAAACAGAGCGCATCATGCGTGAGTACAAGCTAGTAGTTATTGGTTCAGGAGGCGTGGGGAAGTCTGCTCTGACAGTTCAGTTTGTTCAGGGA**

GtF2h1b **~~~~~~~~~~~~~~~~~~~~~~~~~~~~~~~~~~~~~~~~~~~~~~~~~~~~~~~~~~~~~~~~~~~~~~~~~~~~~~~~~~~~~~~~~~~~~~~~~~~~~~~~~~~~~~~~~~~~~~~~**

Rap1A cDNA **GCGCCGGGCCGCGGGATTGTCAATATTTAAACAGAGCGCATCATGCGTGAGTACAAGCTAGTAGTCCTTGGTTCAGGAGGCGTGGGGAAGTCTGCTCTGACAGTTCAGTTTGTTCAGGGG**

2290 2300 2310 2320 2330 2340 2350 2360 2370 2380 2390 2400

....|....|....|....|....|....|....|....|....|....|....|....|....|....|....|....|....|....|....|....|....|....|....|....|

Rap1A-retro1 cDNA **ATTTTTGTTGAAAAATATGACCCAACAATAGAAGATTCCTACAGAAAACAAGTCGAGGTAGATTGCCAACAGTGCATGCTGGAGATCCTGGACACTGTAGGAACTGAGCAATTTACAGCA**

Rap1A-retro1 NCBI **ATTTTTGTTGAAAAATATGACCCAACAATAGAAGATTCCTACAGAAAACAAGTCGAGGTAGATTGCCAACAGTGCATGCTGGAGATCCTGGACACTGTAGGAACTGAGCAATTTACAGCA**

Gtf2h1a **~~~~~~~~~~~~~~~~~~~~~~~~~~~~~~~~~~~~~~~~~~~~~~~~~~~~~~~~~~~~~~~~~~~~~~~~~~~~~~~~~~~~~~~~~~~~~~~~~~~~~~~~~~~~~~~~~~~~~~~~**

Rap1A-retro2 cDNA **ATTTTTGTTGAAAAATATGACCCAATGATAGAAGATTCCTACAGAAAACAAGTCGAGGTAGATTGCCAACAGTGCATGCTGGAGATCCTGGACACTGCAGGAACTGAGCAATTTACAGCA**

Rap1A-retro2 csm **ATTTTTGTTGAAAAATATGACCCAATGATAGAAGATTCCTACAGAAAACAAGTCGAGGTAGATTGCCAACAGTGCATGCTGGAGATCCTGGACACTGCAGGAACTGAGCAATTTACAGCA**

Rap1A-retro2 cel **ATTTTTGTTGAAAAATATGACCCAATGATAGAAGATTCCTACAGAAAACAAGTCGAGGTAGATTGCCAACAGTGCATGCTGGAGATCCTGGACACTGCAGGAACTGAGCAATTTACAGCA**

GtF2h1b **~~~~~~~~~~~~~~~~~~~~~~~~~~~~~~~~~~~~~~~~~~~~~~~~~~~~~~~~~~~~~~~~~~~~~~~~~~~~~~~~~~~~~~~~~~~~~~~~~~~~~~~~~~~~~~~~~~~~~~~~**

Rap1A cDNA **ATTTTTGTTGAAAAATATGACCCAACGATAGAAGATTCCTACAGAAAGCAAGTCGAGGTAGATTGCCAACAGTGCATGCTGGAGATCCTGGACACTGCAGGAACCGAGCAATTTACAGCA**

2410 2420 2430 2440 2450 2460 2470 2480 2490 2500 2510 2520

....|....|....|....|....|....|....|....|....|....|....|....|....|....|....|....|....|....|....|....|....|....|....|....|

Rap1A-retro1 cDNA **ATGAGGGATTTGTATATGAAAAATGGCCAAGGGTTTGCACTAGTTTATTCAATTACAGCTCAGTCTACGTTTAATGATTTACAAGACTTGAGAGAACAGATTTT~ACGGGTTAAAGACAC**

Rap1A-retro1 NCBI **ATGAGGGATTTGTATATGAAAAATGGCCAAGGGTTTGCACTAGTTTATTCAATTACAGCTCAGTCTACGTTTAATGATTTACAAGACTTGAGAGAACAGATTTT~ACGGGTTAAAGACAC**

Gtf2h1a **~~~~~~~~~~~~~~~~~~~~~~~~~~~~~~~~~~~~~~~~~~~~~~~~~~~~~~~~~~~~~~~~~~~~~~~~~~~~~~~~~~~~~~~~~~~~~~~~~~~~~~~~~~~~~~~~~~~~~~~~**

Rap1A-retro2 cDNA **ATGAGGGATTTGTATATGAAGAATGGCCAAGGGTTTGCACTAGTTTATTCAATTACAGCTCAGTCTACGTTTAATGATTTACAGGACGTGAGAGAACAGATTTT~ACGGGTTAAAGACAC**

Rap1A-retro2 csm **ATGAGGGATTTGTATATGAAGAATGGCCAAGGGTTTGCACTAGTTTATTCAATTACAGCTCAGTCTACGTTTAATGATTTACAGGACGTGAGAGAACAGATTTT~ACGGGTTAAAGACAC**

Rap1A-retro2 cel **ATGAGGGATTTGTATATGAAGAATGGCCAAGGGTTTGCACTAGTTTATTCAATTACAGCTCAGTCTACGTTTAATGATTTACAGGACGTGAGAGAACAGATTTTCACGGGTTAAAGACAC**

GtF2h1b **~~~~~~~~~~~~~~~~~~~~~~~~~~~~~~~~~~~~~~~~~~~~~~~~~~~~~~~~~~~~~~~~~~~~~~~~~~~~~~~~~~~~~~~~~~~~~~~~~~~~~~~~~~~~~~~~~~~~~~~~**

Rap1A cDNA **ATGAGGGATTTGTATATGAAGAATGGCCAAGGGTTTGCACTAGTTTATTCAATTACAGCTCAGTCTACGTTTAATGATTTACAAGACTTGAGAGAACAGATTTT~ACGGGTTAAAGACAC**

2530 2540 2550 2560 2570 2580 2590 2600 2610 2620 2630 2640

....|....|....|....|....|....|....|....|....|....|....|....|....|....|....|....|....|....|....|....|....|....|....|....|

Rap1A-retro1 cDNA **AGAAGATGTTCCAATGATTTTGGTTGGCAATAAATGTGACTTGGAAGATGAGCGGGTAGTTGGCAAAGAACAAGGCCAGAATTTAGCAAGACAGTGGTGTAACTGTGCCTTTTTAGAGTC**

Rap1A-retro1 NCBI **AGAAGATGTTCCAATGATTTTGGTTGGCAATAAATGTGACTTGGAAGATGAGCGGGTAGTTGGCAAAGAACAAGGCCAGAATTTAGCAAGACAGTGGTGTAACTGTGCCTTTTTAGAGTC**

Gtf2h1a **~~~~~~~~~~~~~~~~~~~~~~~~~~~~~~~~~~~~~~~~~~~~~~~~~~~~~~~~~~~~~~~~~~~~~~~~~~~~~~~~~~~~~~~~~~~~~~~~~~~~~~~~~~~~~~~~~~~~~~~~**

Rap1A-retro2 cDNA **AGAAGATGTTCCAATGATTTTGGTTGGCAATAAATGTGACTTGGAAGATGAGCGGGTAGTTGGCAAAGAACAAGGCCAGAATTTAGCAAGACAGTGGTGTAACTGTGCCTTTTTAGAATC**

Rap1A-retro2 csm **AGAAGATGTTCCAATGATTTTGGTTGGCAATAAATGTGACTTGGAAGATGAGCGGGTAGTTGGCAAAGAACAAGGCCAGAATTTAGCAAGACAGTGGTGTAACTGTGCCTTTTTAGAATC**

Rap1A-retro2 cel **AGAAGATGTTCCAATGATTTTGGTTGGCAATAAATGTGACTTGGAAGATGAGCGGGTAGTTGGCAAAGAA**

GtF2h1b **~~~~~~~~~~~~~~~~~~~~~~~~~~~~~~~~~~~~~~~~~~~~~~~~~~~~~~~~~~~~~~~~~~~~~~~~~~~~~~~~~~~~~~~~~~~~~~~~~~~~~~~~~~~~~~~~~~~~~~~~**

Rap1A cDNA **AGAAGATGTTCCAATGATTTTGGTTGGCAATAAATGTGACTTGGAAGATGAACGGGTAGTTGGCAAAGAACAAGGCCAGAATTTAGCAAGACAGTGGTGTAACTGTGCCTTTTTAGAATC**

2650 2660 2670 2680 2690 2700 2710 2720 2730 2740 2750 2760

....|....|....|....|....|....|....|....|....|....|....|....|....|....|....|....|....|....|....|....|....|....|....|....|

Rap1A-retro1 cDNA **TTCTGCAAAGTCAAAGATCAACGTTAATGAGATATTTTATGACCTGGTCAGACAGATAAATAGAAAAACACCAGTGGAAAAGAAGAAGCCTAAAAAGAAATCATGTTTGCTGCTCTAGAC**

Rap1A-retro1 NCBI **TTCTGCAAAGTCAAAGATCAACGTTAATGAGATATTTTATGACCTGGTCAGACAGATAAATAGAAAAACACCAGTGGAAAAGAAGAAGCCTAAAAAGAAATCATGTTTGCTGCTCTAGAC**

Gtf2h1a **~~~~~~~~~~~~~~~~~~~~~~~~~~~~~~~~~~~~~~~~~~~~~~~~~~~~~~~~~~~~~~~~~~~~~~~~~~~~~~~~~~~~~~~~~~~~~~~~~~~~~~~~~~~~~~~~~~~~~~~~**

Rap1A-retro2 cDNA **TTCTGCAAAGTCAAAGATCAACGTTAATGAGATATTTTATGACCTGGTCAGACAGATAAATAGAAAAACACCAGTGGAAAAGAAGAAGCCTAAAAAGAAATCATGTTTGCTGCTCTAGAC**

Rap1A-retro2 csm **TTCTGCAAAGTCAAAGATCAACGTTAATGAGATATTTTATGACCTGGTCAGACAGATAAATAGAAAAACACCAGTGGAAAAGAAGAAGCCTAAAAAGAAATCATGTTTGCTGCTCTAGAC**

Rap1A-retro2 cel

GtF2h1b **~~~~~~~~~~~~~~~~~~~~~~~~~~~~~~~~~~~~~~~~~~~~~~~~~~~~~~~~~~~~~~~~~~~~~~~~~~~~~~~~~~~~~~~~~~~~~~~~~~~~~~~~~~~~~~~~~~~~~~~~**

Rap1A cDNA **TTCTGCAAAGTCAAAGATCAACGTTAATGAGATATTTTATGACCTGGTCAGACAGATAAATAGAAAAACACCAGTGGAAAAGAAGAAGCCTAAAAAGAAATCATGTTTGCTGCTCTAGAC**

2770 2780 2790 2800 2810 2820 2830 2840 2850 2860 2870 2880

....|....|....|....|....|....|....|....|....|....|....|....|....|....|....|....|....|....|....|....|....|....|....|....|

Rap1A-retro1 cDNA **CTGAAGTCAGCAGCAGCTCTGAGCCAGATTACAGAAATGAAGAACTGTTGCCTAATTGGAAAGTGCCAGCATTCCAGACTTCAAAAACGAAATCTGAAGAGGCTTCTCCTGTTTTATATA**

Rap1A-retro1 NCBI **CTGAAGTCAGCAGCAGCTCTGAGCCAGATTACAGAAATGAAGAACTGTTGCCTAATTGGAAAGTGCCAGCATTCCAGACTTCAAAAACGAAATCTGAAGAGGCTTCTCCTGTTTTATATA**

Gtf2h1a **~~~~~~~~~~~~~~~~~~~~~~~~~~~~~~~~~~~~~~~~~~~~~~~~~~~~~~~~~~~~~~~~~~~~~~~~~~~~~~~~~~~~~~~~~~~~~~~~~~~~~~~~~~~~~~~~~~~~~~~~**

Rap1A-retro2 cDNA **CTGAAGTCAGCAGCAGCTCTGAGCCAGATTACAGAAATGAAGAGCTGTTGCCTAATTGGAAAGTGCCAGCATTCCAGACTTCAAAAACGAAATCTGAAGAGGCTTCTCCTGTTTTATATA**

Rap1A-retro2 csm **CTGAAGTCAGCAGCAGCTCTGAGCCAGATTACAGAAATGAAGAGCTGTTGCCTAATTGGAAAGTGCCAGCATTCCAGACTTCAAAAACGAAATCTGAAGAGGCTTCTCCTGTTTTATATA**

Rap1A-retro2 cel

GtF2h1b **~~~~~~~~~~~~~~~~~~~~~~~~~~~~~~~~~~~~~~~~~~~~~~~~~~~~~~~~~~~~~~~~~~~~~~~~~~~~~~~~~~~~~~~~~~~~~~~~~~~~~~~~~~~~~~~~~~~~~~~~**

Rap1A cDNA **CTGAAGTCAGCAGCAGCTCTGAGCCAGATTACAGAAATGAAGAACTGTTGCCTAATTGGAAAGTGCCAGCATTCCAGACTTCAAAAACGAAATCTGAAGAGGCTTCTCCTGTTTTATATA**

2890 2900 2910 2920 2930 2940 2950 2960 2970 2980 2990 3000

....|....|....|....|....|....|....|....|....|....|....|....|....|....|....|....|....|....|....|....|....|....|....|....|

Rap1A-retro1 cDNA **TTATGTGAAGAATTTAGATCTTATATTGGTTTGCACAAGTTCCCTGGAGACAAAGTTGCTCTGTGTATATCTCTTGGAAAGAAGACATAGTATTTCTCCTTTGCAATAGCAGTTATAACA**

Rap1A-retro1 NCBI **TTATGTGAAGAATTTAGATCTTATATTGGTTTGCACAAGTTCCCTGGAGACAAAGTTGCTCTGTGTATATCTCTTGGAAAGAAGACATAGTATTTCTCCTTTGCAATAGCAGTTATAACA**

Gtf2h1a **~~~~~~~~~~~~~~~~~~~~~~~~~~~~~~~~~~~~~~~~~~~~~~~~~~~~~~~~~~~~~~~~~~~~~~~~~~~~~~~~~~~~~~~~~~~~~~~~~~~~~~~~~~~~~~~~~~~~~~~~**

Rap1A-retro2 cDNA **TTATGTGAAGAATTTAGATCTTATATTGGTTTGCACAAGTTCCCTGGAGACAAAGTTGCTCTGTGTATATCTCTTGGAAATAAGACATAGTATTTCTCCTTTGCAATAGCAGTTACAACA**

Rap1A-retro2 csm **TTATGTGAAGAATTTAGATCTTATATTGGTTTGCACAAGTTCCCTGGAGACAAAGTTGCTCTGTGTATATCTCTTGGAAATAAGACATAGTATTTCTCCTTTGCAATAGCAGTTACAACA**

Rap1A-retro2 cel

GtF2h1b **~~~~~~~~~~~~~~~~~~~~~~~~~~~~~~~~~~~~~~~~~~~~~~~~~~~~~~~~~~~~~~~~~~~~~~~~~~~~~~~~~~~~~~~~~~~~~~~~~~~~~~~~~~~~~~~~~~~~~~~~**

Rap1A cDNA **TTATGTGAAGAATTTAGATCTTATATTGGTTTGCACAAGTTCCCTGGAGACAAAGTTGCTCTGTGTATATCTCTTGGAAATAAGACATAGTATTTCTCCTTTGCAATAGCAGTTATAACA**

3010 3020 3030 3040 3050 3060 3070 3080 3090 3100 3110 3120

....|....|....|....|....|....|....|....|....|....|....|....|....|....|....|....|....|....|....|....|....|....|....|....|

Rap1A-retro1 cDNA **GATGTGAAATAAGATACTTGACTCTAATACAATTATACAGAAGAGCATGGATGCATTTCAAATGTTAAATGTACTACTATAATCAAATGATTTCATATTGACGTTTTTATCATGACTCCT**

Rap1A-retro1 NCBI **GATGTGAAATAAGATACTTGACTCTAATACAATTATACAGAAGAGCATGGATGCATTTCAAATGTTAAATGTACTACTATAATCAAATGATTTCATATTGACGTTTTTATCATGACTCCT**

Gtf2h1a **~~~~~~~~~~~~~~~~~~~~~~~~~~~~~~~~~~~~~~~~~~~~~~~~~~~~~~~~~~~~~~~~~~~~~~~~~~~~~~~~~~~~~~~~~~~~~~~~~~~~~~~~~~~~~~~~~~~~~~~~**

Rap1A-retro2 cDNA **GATGTGAAATAAGATACTTGACTCTAATACAATTATACAGAAGAGCATGGATGCATTTCAAATGTTAGATGTACTACTATAATCAAATGATTTCATATTGACATTTTTATCATGACTCCT**

Rap1A-retro2 csm **GATGTGAAATAAGATACTTGACTCTAATACAATTATACAGAAGAGCATGGATGCATTTCAAATGTTAGATGTACTACTATAATCAAATGATTTCATATTGACATTTTTATCATGACTCCT**

Rap1A-retro2 cel

GtF2h1b **~~~~~~~~~~~~~~~~~~~~~~~~~~~~~~~~~~~~~~~~~~~~~~~~~~~~~~~~~~~~~~~~~~~~~~~~~~~~~~~~~~~~~~~~~~~~~~~~~~~~~~~~~~~~~~~~~~~~~~~~**

Rap1A cDNA **GATGTGAAATAAGATACTTGACTCTAATACAATTATACAGAAGAGCATGGATGCATTTCAAATGTTAGATGTACTACTATAATCAAATGATTTCATATTGACGTTTTTATCATGACTCCT**

3130 3140 3150 3160 3170 3180 3190 3200 3210 3220 3230 3240

....|....|....|....|....|....|....|....|....|....|....|....|....|....|....|....|....|....|....|....|....|....|....|....|

Rap1A-retro1 cDNA **CCCTGTCAAGCACTAAAAAATTGAACCATCATACTTTATATCTGTAATGATATAGATTATGAAATCTCCTCTCAAACTCATTGCAGCAGATAACTTTTTTGAGTCATTGACTTCATTTTA**

Rap1A-retro1 NCBI **CCCTGTCAAGCACTAAAAAATTGAACCATCATACTTTATATCTGTAATGATATAGATTATGAAATCTCCTCTCAAACTCATTGCAGCAGATAACTTTTTTGAGTCATTGACTTCATTTTA**

Gtf2h1a **~~~~~~~~~~~~~~~~~~~~~~~~~~~~~~~~~~~~~~~~~~~~~~~~~~~~~~~~~~~~~~~~~~~~~~~~~~~~~~~~~~~~~~~~~~~~~~~~~~~~~~~~~~~~~~~~~~~~~~~~**

Rap1A-retro2 cDNA **CCCTGTCAAGCACTAAAAAACTGAACCATCATACTTTATATCTGTAATGATATAGATTATGAAATCTCCTCTCAAACTCATTGCAGCAGATAACTTTTTTGAGTCATTGACTTCATTTTA**

Rap1A-retro2 csm **CCCTGTCAAGCACTAAAAAACTGAACCATCATACTTTATATCTGTAATGATATAGATTATGAAATCTCCTCTCAAACTCATTGCAGCAGATAACTTTTTTGAGTCATTGACTTCATTTTA**

Rap1A-retro2 cel

GtF2h1b **~~~~~~~~~~~~~~~~~~~~~~~~~~~~~~~~~~~~~~~~~~~~~~~~~~~~~~~~~~~~~~~~~~~~~~~~~~~~~~~~~~~~~~~~~~~~~~~~~~~~~~~~~~~~~~~~~~~~~~~~**

Rap1A cDNA **CCCTGTCAAGCACTAAAAAATTGAACCATCATACTTTATATCTGTAATGATATAGATTATGAAATCTCCTCTCAAACTCATTGCAGCAGATAACTTTTTTGAGTCATTGACTTCATTTTA**

3250 3260 3270 3280 3290 3300 3310 3320 3330 3340 3350 3360

....|....|....|....|....|....|....|....|....|....|....|....|....|....|....|....|....|....|....|....|....|....|....|....|

Rap1A-retro1 cDNA **TATTTAAAATTATGAAAATATCATCTGTCATTATATTCTAATTAAAATT~~~~GTGCATGATGCTTTGGAAAAAATGGGTCTTTAATAGGGAAAAACTGGGATAACTGATTTCTATGGC**

Rap1A-retro1 NCBI **TATTTAAAATTATGAAAATATCATCTGTCATTATATTCTAATTAAAATT~~~~GTGCATGATGCTTTGGAAAAAATGGGTCTTTAATAGGGAAAAACTGGGATAACTGATTTCTATGGCT**

Gtf2h1a **~~~~~~~~~~~~~~~~~~~~~~~~~~~~~~~~~~~~~~~~~~~~~~~~~~~~~~~~~~~~~~~~~~~~~~~~~~~~~~~~~~~~~~~~~~~~~~~~~~~~~~~~~~~~~~~~~~~~~~~~**

Rap1A-retro2 cDNA **TATTTAAAATTATGAAAATATCATCTGTCATTATATTCTAATTAAAATTGTTTGTGCATGATGCTCTGGAAAAA~TGGGTCTTTAATAGGGAAAAACTGGGATAACTGATTTCTATGGC**

Rap1A-retro2 csm **TATTTAAAATTATGAAAATATCATCTGTCATTATATTCTAATTAAAATTGTTTGTGCATGATGCTCTGGAAAAA~TGGGTCTTTAATAGGGAAAAACTGGGATAACTGATTTCTATGGCT**

Rap1A-retro2 cel

GtF2h1b **~~~~~~~~~~~~~~~~~~~~~~~~~~~~~~~~~~~~~~~~~~~~~~~~~~~~~~~~~~~~~~~~~~~~~~~~~~~~~~~~~~~~~~~~~~~~~~~~~~~~~~~~~~~~~~~~~~~~~~~~**

Rap1A cDNA **TATTTAAAATTATGAAAATATCATCTGTCATTATATTCTAATTAAAATTGTTTGTGCATAATGCTTTGGAAAAA~TGGGTCTTTTATAGGGAAAAACTGGGATAACTGATTTCTATGGCT**

3370 3380 3390 3400 3410 3420 3430 3440 3450 3460 3470 3480

....|....|....|....|....|....|....|....|....|....|....|....|....|....|....|....|....|....|....|....|....|....|....|....|

Rap1A-retro1 cDNA

Rap1A-retro1 NCBI **TTCAAAGCTAGAATATATAATATACTAAACCAACTCTAATATTGTTTTTTGTGTTTTACTGTCAGATTAAATCACAGCTTTTATGGATGATTAAATTTAAAAAAGAAAAAAGAAAAACCA**

Gtf2h1a **~~~~~~~~~~~~~~~~~~~~~~~~~~~~~~~~~~~~~~~~~~~~~~~~~~~~~~~~~~~~~~~~~~~~~~~~~~~~~~~~~~~~~~~~~~~~~~~~~~~~~~~~~~~~~~~~~~~~~~~~**

Rap1A-retro2 cDNA

Rap1A-retro2 csm **TTCAAAGCTAGAATATATAATATACTAAACCAACTTTAATATTGCTTTTTGTGTTTTAATGTCAGATTAAATTACAGCTTTTATGGATGATTAAATTTAAAAAA**

Rap1A-retro2 cel

GtF2h1b **~~~~~~~~~~~~~~~~~~~~~~~~~~~~~~~~~~~~~~~~~~~~~~~~~~~~~~~~~~~~~~~~~~~~~~~~~~~~~~~~~~~~~~~~~~~~~~~~~~~~~~~~~~~~~~~~~~~~~~~~**

Rap1A cDNA **TTCAAAGCTAGAATATATAATATACTAAACCAACTCTAATATTGCTTCTTGTGTTTTACTGTCAGATTAAATTACAGCTTTTATGGATGATTAAATTTTAGTACATTTTCAAAAAAAAAA**

3490 3500 3510 3520 3530 3540 3550 3560 3570 3580 3590 3600

....|....|....|....|....|....|....|....|....|....|....|....|....|....|....|....|....|....|....|....|....|....|....|....|

Rap1A-retro1 cDNA

Rap1A-retro1 NCBI **GAAGGCCAGAAGAGCTGGCACTGGAGCCAAGCCTAACAGTCTGAGCTTGACCCTACATCCTACTTGGTCTGGAGAGAGAACTGACTCCTACACGTTTCTTCTGAGTGTAGCAAATACACA**

Gtf2h1a **~~~~~~~~~~~~~~~~~~~~~~~~~~~~~~~~~~~~~~~~~~~~~~~~~~~~~~~~~~~~~~~~~~~~~~~~~~~~~~~~~~~~~~~~~~~~~~~~~~~~~~~~~~~~~~~~~~~~~~~~**

Rap1A-retro2 cDNA

Rap1A-retro2 csm

Rap1A-retro2 cel

GtF2h1b **~~~~~~~~~~~~~~~~~~~~~~~~~~~~~~~~~~~~~~~~~~~~~~~~~~~~~~~~~~~~~~~~~~~~~~~~~~~~~~~~~~~~~~~~~~~~~~~~~~~~~~~~~~~~~~~~~~~~~~~~**

Rap1A cDNA **AAAAAAAAAAAAAAAAAAAAAAA**

3610 3620 3630 3640 3650 3660 3670 3680 3690 3700 3710 3720

....|....|....|....|....|....|....|....|....|....|....|....|....|....|....|....|....|....|....|....|....|....|....|....|

Rap1A-retro1 cDNA

Rap1A-retro1 NCBI **CCTCCATCCTGCTTTAACACACTACATAAAATAGAACACAAAGAGAAGCAGTAGAATGATTCTCATAAAGGCTGGAAGGAGGCACTGCTGTGCAGCTGATAGGGCACTGAGAATATTTTC**

Gtf2h1a **~~~~~~~~~~~~~~~~~~~~~~~~~~~~~~~~~~~~~~~~~~~~~~~~~~~~~~~~~~~~~~~~~~~~~~~~~~~~~~~~~~~~~~~~~~~~~~~~~~~~~~~~~~~~~~~~~~~~~~~~**

Rap1A-retro2 cDNA

Rap1A-retro2 csm

Rap1A-retro2 cel

GtF2h1b **~~~~~~~~~~~~~~~~~~~~~~~~~~~~~~~~~~~~~~~~~~~~~~~~~~~~~~~~~~~~~~~~~~~~~~~~~~~~~~~~~~~~~~~~~~~~~~~~~~~~~~~~~~~~~~~~~~~~~~~~**

Rap1A cDNA

3730 3740 3750 3760 3770 3780 3790 3800 3810 3820 3830 3840

....|....|....|....|....|....|....|....|....|....|....|....|....|....|....|....|....|....|....|....|....|....|....|....|

Rap1A-retro1 cDNA

Rap1A-retro1 NCBI **ACTAACTGGCAAGTACTTTTTTTGGAACAGATCTCATTATGTAACTGGGGCTAGTGTAGTGAGAATTCTGGAATTTTGGTTTCTAAAAAAATAAAAAAAACAAACAAACAACAACAACAA**

Gtf2h1a **~~~~~~~~~~~~~~~~~~~~~~~~~~~~~~~~~~~~~~~~~~~~~~~~~~~~~~~~~~~~~~~~~~~~~~~~~~~~~~~~~~~~~~~~~~~~~~~~~~~~~~~~~~~~~~~~~~~~~~~~**

Rap1A-retro2 cDNA

Rap1A-retro2 csm

Rap1A-retro2 cel

GtF2h1b **~~~~~~~~~~~~~~~~~~~~~~~~~~~~~~~~~~~~~~~~~~~~~~~~~~~~~~~~~~~~~~~~~~~~~~~~~~~~~~~~~~~~~~~~~~~~~~~~~~~~~~~~~~~~~~~~~~~~~~~~**

Rap1A cDNA

3850 3860 3870 3880 3890 3900 3910 3920 3930 3940 3950 3960

....|....|....|....|....|....|....|....|....|....|....|....|....|....|....|....|....|....|....|....|....|....|....|....|

Rap1A-retro1 cDNA

Rap1A-retro1 NCBI **CAACAACACCCAAACCAACCAACCAACCAAACAAACAAAAAAACCCCAGAAATATTATAGGAATATCTTTTTGTTTTAAGTGCAAAACCTGGCTGCTCTGCCTTAGCTAACTGATTTGCC**

Gtf2h1a **~~~~~~~~~~~~~~~~~~~~~~~~~~~~~~~~~~~~~~~~~~~~~~~~~~~~~~~~~~~~~~~~~~~~~~~~~~~~~~~~~~~~~~~~~~~~~~~~~~~~~~~~~~~~~~~~~~~~~~~~**

Rap1A-retro2 cDNA

Rap1A-retro2 csm

Rap1A-retro2 cel

GtF2h1b **~~~~~~~~~~~~~~~~~~~~~~~~~~~~~~~~~~~~~~~~~~~~~~~~~~~~~~~~~~~~~~~~~~~~~~~~~~~~~~~~~~~~~~~~~~~~~~~~~~~~~~~~~~~~~~~~~~~~~~~~**

Rap1A cDNA

3970 3980 3990 4000 4010 4020 4030 4040 4050 4060 4070 4080

....|....|....|....|....|....|....|....|....|....|....|....|....|....|....|....|....|....|....|....|....|....|....|....|

Rap1A-retro1 cDNA

Rap1A-retro1 NCBI **TCATGCTCTAACGGAGGCACGGTTTCACCAACTGCAGATATAGTTTCCAAGACTGTGATGTTGGAATTCTGGCAACTTTTCATATGGTATATAAATGTTTGGGAAGGCCTGAGAGGTGGG**

Gtf2h1a **~~~~~~~~~~~~~~~~~~~~~~~~~~~~~~~~~~~~~~~~~~~~~~~~~~~~~~~~~~~~~~~~~~~~~~~~~~~~~~~~~~~~~~~~~~~~~~~~~~~~~~~~~~~~~~~~~~~~~~~~**

Rap1A-retro2 cDNA

Rap1A-retro2 csm

Rap1A-retro2 cel

GtF2h1b **~~~~~~~~~~~~~~~~~~~~~~~~~~~~~~~~~~~~~~~~~~~~~~~~~~~~~~~~~~~~~~~~~~~~~~~~~~~~~~~~~~~~~~~~~~~~~~~~~~~~~~~~~~~~~~~~~~~~~~~~**

Rap1A cDNA

4090 4100 4110 4120 4130 4140 4150 4160 4170 4180 4190 4200

....|....|....|....|....|....|....|....|....|....|....|....|....|....|....|....|....|....|....|....|....|....|....|....|

Rap1A-retro1 cDNA

Rap1A-retro1 NCBI **GTAGGGGTTGGCTGTGGTTTGTTAGTAGTCATGCTCAAGAAAAAAAGACACTAGATTCAGAGACCTCTATCTCTCTCTCTCCCATCTAGTGATAGGGAGTAAAATTGGGGAGAAAGGGGA**

Gtf2h1a **~~~~~~~~~~~~~~~~~~~~~~~~~~~~~~~~~~~~~~~~~~~~~~~~~~~~~~~~~~~~~~~~~~~~~~~~~~~~~~~~~~~~~~~~~~~~~~~~~~~~~~~~~~~~~~~~~~~~~~~~**

Rap1A-retro2 cDNA

Rap1A-retro2 csm

Rap1A-retro2 cel

GtF2h1b **~~~~~~~~~~~~~~~~~~~~~~~~~~~~~~~~~~~~~~~~~~~~~~~~~~~~~~~~~~~~~~~~~~~~~~~~~~~~~~~~~~~~~~~~~~~~~~~~~~~~~~~~~~~~~~~~~~~~~~~~**

Rap1A cDNA

4210 4220 4230 4240 4250 4260 4270 4280 4290 4300 4310 4320

....|....|....|....|....|....|....|....|....|....|....|....|....|....|....|....|....|....|....|....|....|....|....|....|

Rap1A-retro1 cDNA

Rap1A-retro1 NCBI **GATAAAAGGGTGGGAAAAATAAGAACTCACAAAGCAGCAAAGGCCAGCTACAGATTAGCTTAAGTACACATACTTTATATACTCTTTTGAATGCAGTGAAGGTTTTAAAATGAAAAATAG**

Gtf2h1a **~~~~~~~~~~~~~~~~~~~~~~~~~~~~~~~~~~~~~~~~~~~~~~~~~~~~~~~~~~~~~~~~~~~~~~~~~~~~~~~~~~~~~~~~~~~~~~~~~~~~~~~~~~~~~~~~~~~~~~~~**

Rap1A-retro2 cDNA

Rap1A-retro2 csm

Rap1A-retro2 cel

GtF2h1b **~~~~~~~~~~~~~~~~~~~~~~~~~~~~~~~~~~~~~~~~~~~~~~~~~~~~~~~~~~~~~~~~~~~~~~~~~~~~~~~~~~~~~~~~~~~~~~~~~~~~~~~~~~~~~~~~~~~~~~~~**

Rap1A cDNA

4330 4340 4350 4360 4370 4380 4390 4400 4410 4420 4430 4440

....|....|....|....|....|....|....|....|....|....|....|....|....|....|....|....|....|....|....|....|....|....|....|....|

Rap1A-retro1 cDNA

Rap1A-retro1 NCBI **CATCCTGAGCAAAGAAAAGAACAATGTACTCGCATATATGAGAACAAAGTTAATTTCATCATTCAGAAAGAGGCAGACAACTGGGGCTTACATTTGTGCTTAAATACTGCCTCCGAATCT**

Gtf2h1a **~~~~~~~~~~~~~~~~~~~~~~~~~~~~~~~~~~~~~~~~~~~~~~~~~~~~~~~~~~~~~~~~~~~~~~~~~~~~~~~~~~~~~~~~~~~ATTTGTGCTTAAATACTGCCTCCGAATCT**

Rap1A-retro2 cDNA

Rap1A-retro2 csm

Rap1A-retro2 cel

GtF2h1b **~~~~~~~~~~~~~~~~~~~~~~~~~~~~~~~~~~~~~~~~~~~~~~~~~~~~~~~~~~~~~~~~~~~~~~~~~~~~~~~~~~~~~~~~~~~ATTTGTGCTTAAATACTGCCTCCGAATCT**

Rap1A cDNA

4450 4460 4470 4480 4490 4500 4510 4520

....|....|....|....|....|....|....|....|....|....|....|....|....|....|....|....|....|...

Rap1A-retro1 cDNA

Rap1A-retro1 NCBI **TTTCTTGGAATGGGCAGAGCTTTGTAACTTGGAACCGTTCCAAATTACTCTTCATTTTCACTACCTAAAAAAGATGAGAAACAATGAA**

Gtf2h1a **TTTCTTGGAATGGGCAGAGCTTTGTAACTTGGAACCGTTCCAAATTACTCTTCATTTTCACTAC**

Rap1A-retro2 cDNA

Rap1A-retro2 csm

Rap1A-retro2 cel

GtF2h1b **TTTCTTGGAATGGGCAGAGCTTTGTAACTTGGAACCGTTCCAAATTACTCTTCATTTTCACTAC**

Rap1A cDNA
