# Supplementary material for: Retrotransposition and mutation events yield Rap1 GTPases with differential signalling capacity
Source: BMC Evol Biol. 2010 Feb 19;10:55. doi: 10.1186/1471-2148-10-55 (PMC2831893; doi:10.1186/1471-2148-10-55)

**Supplementary Figure 3.** The phylogenetic gene tree depicts the evolution of mammalian Rap1A and Rap1B genes, and mRap1A-retro1, mRap1A-retro2 and hRap1B-retro retrogenes. See Methods for details on tree construction. Numbers at internal edges indicate bootstrap values which were obtained for 100 replicates.


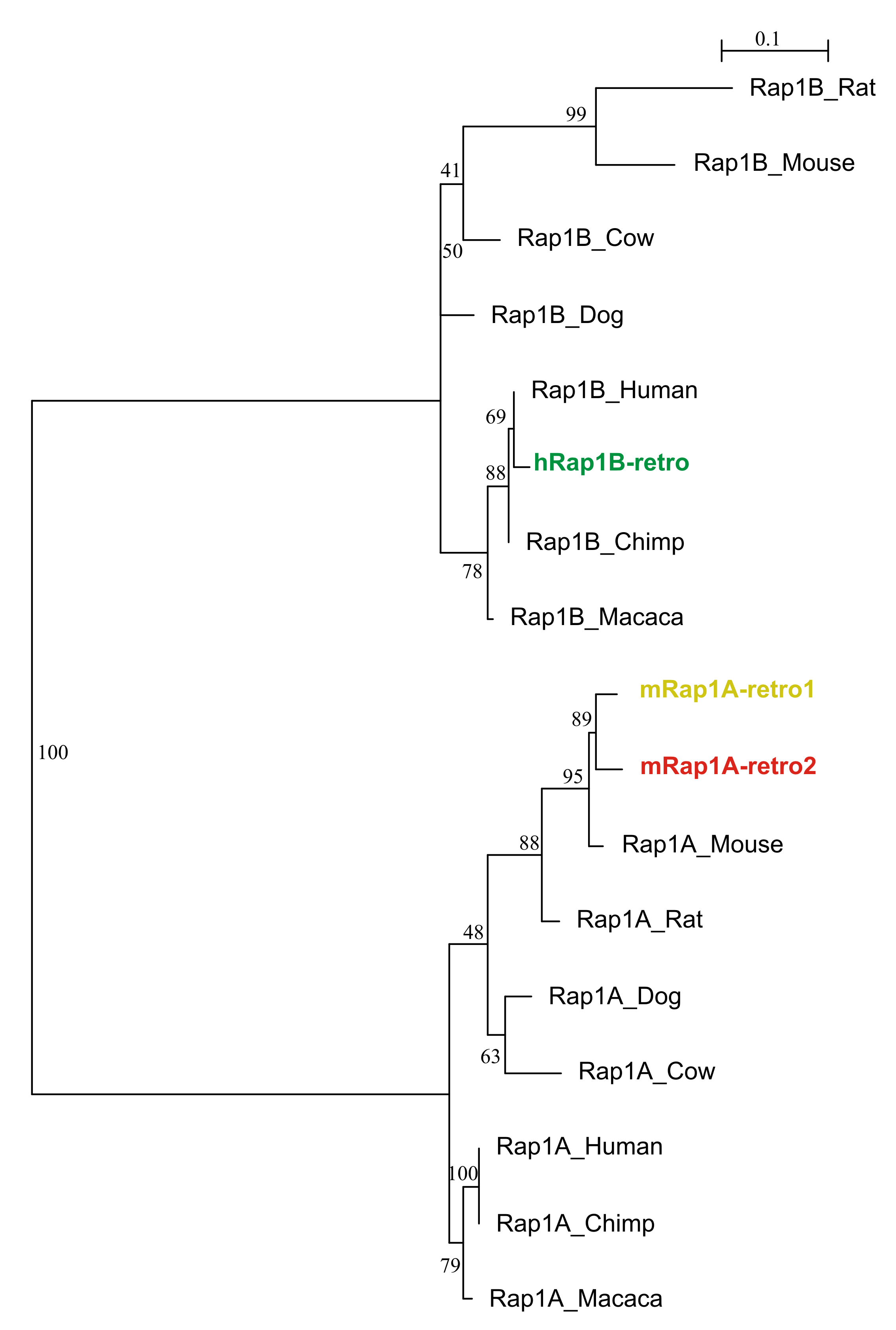

Supplement: Additional file 3 — Supplemental Figure S3. The gene tree for the Rap1 genes. [file 1471-2148-10-55-S3.DOC]
